# Development of a highly differentiated rat brain organoid model for exploring glioblastoma invasion dynamics and therapy

**DOI:** 10.1093/neuonc/noaf271

**Published:** 2025-11-24

**Authors:** Wenjing Zhou, Elena Martinez-Garcia, Katharina Sarnow, Georgia Kanli, Petr V Nazarov, Yaquan Li, Stephanie Gisela Schwab, Johannes Meiser, Christian Jaeger, Jakub Mieczkowski, Agnieszka Misztak, Frits A Thorsen, Konrad Grützmann, Boris Mihaljevic, Barbara Van Loon, Jubayer A Hossain, Yan Zhang, Zhiyi Xue, Wenjie Li, Shannon S Moreino, Anna Golebiewska, Simone P Niclou, Magnar Bjørås, Saverio Tardito, Justin V Joesph, Taral R Lunavat, Halala S Saed, Marzieh Bahador, Mingzhi Han, Carina Fabian, Hrvoje Miletic, Xingang Li, Gunnar Dittmar, Olivier Keunen, Barbara Klink, Jian Wang, Rolf Bjerkvig

**Affiliations:** Department of Blood Transfusion, Shandong Provincial Hospital Affiliated to Shandong First Medical University, Jinan, Shandong, China (W.Z.); Department of Neurosurgery, Qilu Hospital of Shandong University and Brain Science Research Institute, Shandong University, Jinan, Shandong, China (W.Z., Y.L., Y.Z., Z.X., W.L., M.H., X.L., J.W., R.B.); Key Laboratory of Brain Functional Remodeling, Jinan, Shandong, China (W.Z., Y.L., Y.Z., Z.X., W.L., M.H., X.L., J.W., R.B.); NORLUX Neuro-Oncology Laboratory, Department of Biomedicine, University of Bergen, Bergen, Norway (W.Z., K.S., Y.L., S.G.S., F.A.T., J.A.H., S.S.M., S.P.N., J.V.J., T.R.L., H.S.S., M.B., M.H., C.F., H.M., B.K., J.W., R.B.); Proteomics of Cellular Signaling, Department of Infection and Immunity, Luxembourg Institute of Health, Luxembourg, Luxembourg (E.M.-G., G.D.); NORLUX Neuro-Oncology Laboratory, Department of Biomedicine, University of Bergen, Bergen, Norway (W.Z., K.S., Y.L., S.G.S., F.A.T., J.A.H., S.S.M., S.P.N., J.V.J., T.R.L., H.S.S., M.B., M.H., C.F., H.M., B.K., J.W., R.B.); Department of Neurosurgery, Xin Tang Laboratory, Boston Children’s Hospital/Harvard Medical School, Boston, Massachusetts, USA (K.S.); Translational Radiomics, Department of Cancer Research, Luxembourg Institute of Health, Luxembourg, Luxembourg (G.K., O.K.); Multiomics Data Science Group, Department of Cancer Research, Luxembourg Institute of Health, Luxembourg, Luxembourg (P.V.N.); Bioinformatics and AI Unit, Department of Medical Informatics, Luxembourg Institute of Health, Luxembourg, Luxembourg (P.V.N.); Department of Neurosurgery, Qilu Hospital of Shandong University and Brain Science Research Institute, Shandong University, Jinan, Shandong, China (W.Z., Y.L., Y.Z., Z.X., W.L., M.H., X.L., J.W., R.B.); Key Laboratory of Brain Functional Remodeling, Jinan, Shandong, China (W.Z., Y.L., Y.Z., Z.X., W.L., M.H., X.L., J.W., R.B.); NORLUX Neuro-Oncology Laboratory, Department of Biomedicine, University of Bergen, Bergen, Norway (W.Z., K.S., Y.L., S.G.S., F.A.T., J.A.H., S.S.M., S.P.N., J.V.J., T.R.L., H.S.S., M.B., M.H., C.F., H.M., B.K., J.W., R.B.); NORLUX Neuro-Oncology Laboratory, Department of Biomedicine, University of Bergen, Bergen, Norway (W.Z., K.S., Y.L., S.G.S., F.A.T., J.A.H., S.S.M., S.P.N., J.V.J., T.R.L., H.S.S., M.B., M.H., C.F., H.M., B.K., J.W., R.B.); Cancer Metabolism Group, Department of Cancer Research, Luxembourg Institute of Health, Luxembourg, Luxembourg (J.M.); Luxembourg Centre for Systems Biomedicine (LCSB), University of Luxembourg, Luxembourg, Luxembourg (C.J.); 3P-Medicine Laboratory, Medical University of Gdańsk, Gdańsk, Poland (J.M., A.M.); 3P-Medicine Laboratory, Medical University of Gdańsk, Gdańsk, Poland (J.M., A.M.); NORLUX Neuro-Oncology Laboratory, Department of Biomedicine, University of Bergen, Bergen, Norway (W.Z., K.S., Y.L., S.G.S., F.A.T., J.A.H., S.S.M., S.P.N., J.V.J., T.R.L., H.S.S., M.B., M.H., C.F., H.M., B.K., J.W., R.B.); Molecular Imaging Center, Department of Biomedicine, University of Bergen, Bergen, Norway (F.A.T.); Core Unit for Molecular Tumour Diagnostics (CMTD), National Center for Tumour Diseases (NCT), Dresden, Germany (K.G., B.K.); Institute for Medical Informatics and Biometry, Medical Faculty, TU Dresden, Dresden, Germany (K.G.); Department of Clinical and Molecular Medicine, Norwegian University of Science and Technology, Trondheim, Norway (B.M., B.V.L., M.B.); Department of Microbiology and Parasitology, University Hospital of Split, Split, Croatia (B.M.); Department of Clinical and Molecular Medicine, Norwegian University of Science and Technology, Trondheim, Norway (B.M., B.V.L., M.B.); NORLUX Neuro-Oncology Laboratory, Department of Biomedicine, University of Bergen, Bergen, Norway (W.Z., K.S., Y.L., S.G.S., F.A.T., J.A.H., S.S.M., S.P.N., J.V.J., T.R.L., H.S.S., M.B., M.H., C.F., H.M., B.K., J.W., R.B.); Faculty of Nursing and Health Sciences, Nord University, Namsos, Norway (J.A.H.); Department of Neurosurgery, Qilu Hospital of Shandong University and Brain Science Research Institute, Shandong University, Jinan, Shandong, China (W.Z., Y.L., Y.Z., Z.X., W.L., M.H., X.L., J.W., R.B.); Key Laboratory of Brain Functional Remodeling, Jinan, Shandong, China (W.Z., Y.L., Y.Z., Z.X., W.L., M.H., X.L., J.W., R.B.); Department of Neurosurgery, Qilu Hospital of Shandong University and Brain Science Research Institute, Shandong University, Jinan, Shandong, China (W.Z., Y.L., Y.Z., Z.X., W.L., M.H., X.L., J.W., R.B.); Key Laboratory of Brain Functional Remodeling, Jinan, Shandong, China (W.Z., Y.L., Y.Z., Z.X., W.L., M.H., X.L., J.W., R.B.); Department of Neurosurgery, Qilu Hospital of Shandong University and Brain Science Research Institute, Shandong University, Jinan, Shandong, China (W.Z., Y.L., Y.Z., Z.X., W.L., M.H., X.L., J.W., R.B.); Key Laboratory of Brain Functional Remodeling, Jinan, Shandong, China (W.Z., Y.L., Y.Z., Z.X., W.L., M.H., X.L., J.W., R.B.); NORLUX Neuro-Oncology Laboratory, Department of Biomedicine, University of Bergen, Bergen, Norway (W.Z., K.S., Y.L., S.G.S., F.A.T., J.A.H., S.S.M., S.P.N., J.V.J., T.R.L., H.S.S., M.B., M.H., C.F., H.M., B.K., J.W., R.B.); NORLUX Neuro-Oncology Laboratory, Department of Cancer Research, Luxembourg Institute of Health, Luxembourg, Luxembourg (A.G., S.P.N., C.F.); NORLUX Neuro-Oncology Laboratory, Department of Biomedicine, University of Bergen, Bergen, Norway (W.Z., K.S., Y.L., S.G.S., F.A.T., J.A.H., S.S.M., S.P.N., J.V.J., T.R.L., H.S.S., M.B., M.H., C.F., H.M., B.K., J.W., R.B.); NORLUX Neuro-Oncology Laboratory, Department of Cancer Research, Luxembourg Institute of Health, Luxembourg, Luxembourg (A.G., S.P.N., C.F.); Department of Clinical and Molecular Medicine, Norwegian University of Science and Technology, Trondheim, Norway (B.M., B.V.L., M.B.); Center for Cancer Research, Medical University of Vienna, Comprehensive Cancer Center, Vienna, Austria (S.T.); NORLUX Neuro-Oncology Laboratory, Department of Biomedicine, University of Bergen, Bergen, Norway (W.Z., K.S., Y.L., S.G.S., F.A.T., J.A.H., S.S.M., S.P.N., J.V.J., T.R.L., H.S.S., M.B., M.H., C.F., H.M., B.K., J.W., R.B.); NORLUX Neuro-Oncology Laboratory, Department of Biomedicine, University of Bergen, Bergen, Norway (W.Z., K.S., Y.L., S.G.S., F.A.T., J.A.H., S.S.M., S.P.N., J.V.J., T.R.L., H.S.S., M.B., M.H., C.F., H.M., B.K., J.W., R.B.); NORLUX Neuro-Oncology Laboratory, Department of Biomedicine, University of Bergen, Bergen, Norway (W.Z., K.S., Y.L., S.G.S., F.A.T., J.A.H., S.S.M., S.P.N., J.V.J., T.R.L., H.S.S., M.B., M.H., C.F., H.M., B.K., J.W., R.B.); NORLUX Neuro-Oncology Laboratory, Department of Biomedicine, University of Bergen, Bergen, Norway (W.Z., K.S., Y.L., S.G.S., F.A.T., J.A.H., S.S.M., S.P.N., J.V.J., T.R.L., H.S.S., M.B., M.H., C.F., H.M., B.K., J.W., R.B.); Department of Neurosurgery, Qilu Hospital of Shandong University and Brain Science Research Institute, Shandong University, Jinan, Shandong, China (W.Z., Y.L., Y.Z., Z.X., W.L., M.H., X.L., J.W., R.B.); Key Laboratory of Brain Functional Remodeling, Jinan, Shandong, China (W.Z., Y.L., Y.Z., Z.X., W.L., M.H., X.L., J.W., R.B.); NORLUX Neuro-Oncology Laboratory, Department of Biomedicine, University of Bergen, Bergen, Norway (W.Z., K.S., Y.L., S.G.S., F.A.T., J.A.H., S.S.M., S.P.N., J.V.J., T.R.L., H.S.S., M.B., M.H., C.F., H.M., B.K., J.W., R.B.); NORLUX Neuro-Oncology Laboratory, Department of Biomedicine, University of Bergen, Bergen, Norway (W.Z., K.S., Y.L., S.G.S., F.A.T., J.A.H., S.S.M., S.P.N., J.V.J., T.R.L., H.S.S., M.B., M.H., C.F., H.M., B.K., J.W., R.B.); NORLUX Neuro-Oncology Laboratory, Department of Cancer Research, Luxembourg Institute of Health, Luxembourg, Luxembourg (A.G., S.P.N., C.F.); NORLUX Neuro-Oncology Laboratory, Department of Biomedicine, University of Bergen, Bergen, Norway (W.Z., K.S., Y.L., S.G.S., F.A.T., J.A.H., S.S.M., S.P.N., J.V.J., T.R.L., H.S.S., M.B., M.H., C.F., H.M., B.K., J.W., R.B.); Department of Pathology, Haukeland University Hospital, Bergen, Norway (H.M.); Department of Neurosurgery, Qilu Hospital of Shandong University and Brain Science Research Institute, Shandong University, Jinan, Shandong, China (W.Z., Y.L., Y.Z., Z.X., W.L., M.H., X.L., J.W., R.B.); Key Laboratory of Brain Functional Remodeling, Jinan, Shandong, China (W.Z., Y.L., Y.Z., Z.X., W.L., M.H., X.L., J.W., R.B.); Proteomics of Cellular Signaling, Department of Infection and Immunity, Luxembourg Institute of Health, Luxembourg, Luxembourg (E.M.-G., G.D.); Translational Radiomics, Department of Cancer Research, Luxembourg Institute of Health, Luxembourg, Luxembourg (G.K., O.K.); NORLUX Neuro-Oncology Laboratory, Department of Biomedicine, University of Bergen, Bergen, Norway (W.Z., K.S., Y.L., S.G.S., F.A.T., J.A.H., S.S.M., S.P.N., J.V.J., T.R.L., H.S.S., M.B., M.H., C.F., H.M., B.K., J.W., R.B.); Core Unit for Molecular Tumour Diagnostics (CMTD), National Center for Tumour Diseases (NCT), Dresden, Germany (K.G., B.K.); MGZ—Medical Genetics Center, Munich, Germany (B.K.); Department of Cancer Research, Luxembourg Institute of Health, Luxembourg, Luxembourg (B.K.); Department of Neurosurgery, Qilu Hospital of Shandong University and Brain Science Research Institute, Shandong University, Jinan, Shandong, China (W.Z., Y.L., Y.Z., Z.X., W.L., M.H., X.L., J.W., R.B.); Key Laboratory of Brain Functional Remodeling, Jinan, Shandong, China (W.Z., Y.L., Y.Z., Z.X., W.L., M.H., X.L., J.W., R.B.); NORLUX Neuro-Oncology Laboratory, Department of Biomedicine, University of Bergen, Bergen, Norway (W.Z., K.S., Y.L., S.G.S., F.A.T., J.A.H., S.S.M., S.P.N., J.V.J., T.R.L., H.S.S., M.B., M.H., C.F., H.M., B.K., J.W., R.B.); Department of Neurosurgery, Qilu Hospital of Shandong University and Brain Science Research Institute, Shandong University, Jinan, Shandong, China (W.Z., Y.L., Y.Z., Z.X., W.L., M.H., X.L., J.W., R.B.); Key Laboratory of Brain Functional Remodeling, Jinan, Shandong, China (W.Z., Y.L., Y.Z., Z.X., W.L., M.H., X.L., J.W., R.B.); NORLUX Neuro-Oncology Laboratory, Department of Biomedicine, University of Bergen, Bergen, Norway (W.Z., K.S., Y.L., S.G.S., F.A.T., J.A.H., S.S.M., S.P.N., J.V.J., T.R.L., H.S.S., M.B., M.H., C.F., H.M., B.K., J.W., R.B.)

**Keywords:** brain microenvironment, brain organoids, invasion, therapy

## Abstract

**Background:**

Human brain organoids (BOs) are important models for studying early brain development and neurological disorders. While techniques for creating BOs are advancing, they remain developmental structures. Therefore, when human BOs are used to studying glioma–host interactions, the tumor behavior may be influenced by the BO-developmental microenvironment. Here, we describe the maturation of rat brain organoids (rBOs) into fully differentiated BOs and demonstrate their value as a model for studying glioblastoma (GB)–host interactions and their use in testing therapeutic interventions.

**Materials and Methods:**

rBOs were obtained from fetal cortical brains on the 18th day of gestation. Transcriptomic, proteomic, and metabolomic analyses determined their differentiation into maturity. Their developmental trajectory was compared to human BOs derived from induced pluripotent stem cells as well as to rat brain development. Tumor–rBO interactions, including invasion parameters and therapeutic interactions, were studied using 5 human GB models.

**Results:**

The rBOs develop into organized structures with myelinated neurons, oligodendrocytes, synapses, and glial cells, mirroring the rat brain development. GB invasion in rBOs matched those observed in orthotopic xenografts, enabling real-time assessment of invasion metrics: cellular heterogeneity, single-cell invasion speed, and tumor progression. The BOs had a strong impact on GB transcriptional activity and can be used to study therapeutic interventions. The rBO differentiation status influenced GB invasion capacity.

**Conclusions:**

The rBOs serve as an effective target brain structure for studying GB invasion parameters and for evaluating therapeutic interventions. Their rapid development into mature brain tissue makes rBOs a valuable brain avatar system for studying tumor–host interactions.

## Graphical Abstract

**Graphical Abstract:**
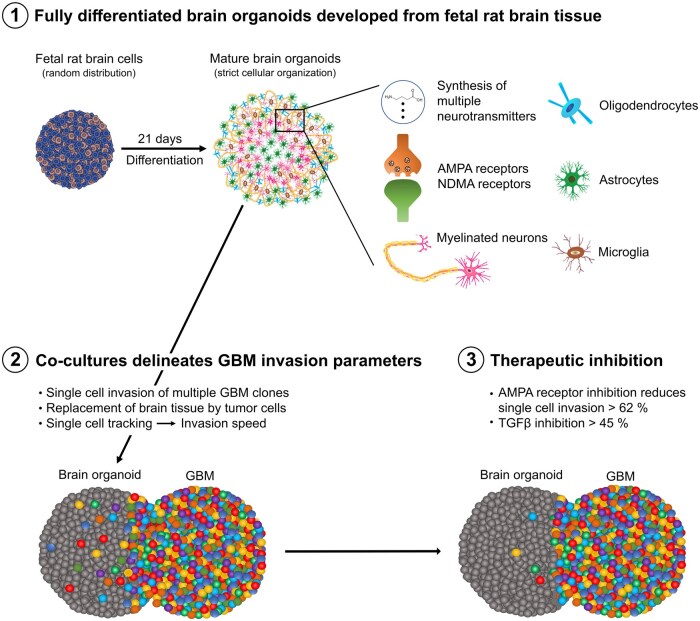

Key Points

- The development of a highly differentiated rat brain organoid model.
- Transcriptomics demonstrates the influence of the brain organoids on GB cells.
- Assessment of central GB invasion parameters.
- The use of the rat brain organoids to study different therapeutic interventions.

Importance of the Study

We describe the development of a highly differentiated rat brain organoid (rBO) model that mirrors the rat brain development. This represents a significant advancement compared to human brain organoid systems that do not reach terminal differentiation. The rBOs contain all major neural cell types at physiological ratios, including myelination and synaptic activity. rBO physiological characteristics were evaluated through transcriptomic, proteomic, and metabolomic profiling. The rBOs were used for studying GB invasion parameters, including the assessment of invasive cellular heterogeneity and automated quantification of both single-cell invasion and tissue replacement. We demonstrate that human GB models display different invasion patterns in the rBOs, reflecting their in vivo behavior. Additionally, the rBOs were used for therapeutic testing, as shown by TGFβ inhibition and AMPA receptor targeting. This physiologically relevant platform addresses key limitations of previous models and offers significant potential for evaluating new therapeutic strategies in the field of neuro-oncology.

Human brain organoids (hBOs) derived from pluripotent stem cells are powerful models for studying brain development and the mechanisms of neurological diseases.^1^ They replicate key features of the developing human brain through their structural organization and cellular diversity,^2^ yet terminal differentiation is not achieved. This is partly due to inherent temporal constraints, as human brain development takes years, while hBOs are grown over much shorter periods.^3^^,^^4^ For example, myelination that happens during infancy and early childhood,^5^ is missing in hBOs unless specialized organoid protocols are used.^6^ Additionally, despite the advances in co-culture techniques involving various supporting cell types to improve differentiation and functionality, fully mature hBOs have not yet been achieved.^7–11^

An alternative approach involves the generation of BOs as assembloids from fetal brain tissue. While this is not possible in humans, it can be accomplished using rat cortical brain tissue harvested at a precise time point—18 days of gestation (E18). After dissociating and aggregating cortical cells, followed by a 21-day cell culture period, rat brain organoids (rBOs) are established (Supplementary Figure S1A and B).^2^ Although this model was developed in the 1980s, it should be recognized that scientific innovation often emerges from applying cutting-edge technologies to established biological systems. The present work embodies this principle by utilizing modern tools to yield new biological insights into this BO system.

Here, we show that rBOs undergo differentiation into complex and highly specialized brain structures. This is shown by molecular profiling, including transcriptomic, proteomic, and metabolomic analyses, which together provide an in-depth characterization of their developmental state and maturity. We further show how GB adapts to differentiated brain tissue and how important GB invasion parameters can be delineated. In this context, several ex vivo models have been developed to study brain tumor invasion in real-time, including GB invasion into collagen gels, Boyden chambers, brain slices, and brain organoids (BOs),^6^ as well as using intravital microscopy.^12^ hBOs, with their genetic fidelity, have proven effective in studying glioma invasion, providing insights relevant to the disease.^7–11^ A limitation, however, is that these studies involve glioma invasion into developing brain structures, where developmental signaling pathways may affect GB cell behavior.

Using rBOs, we establish a physiologically relevant model that enables real-time tracking of GB invasion, including migration dynamics and cellular heterogeneity. Furthermore, the rBOs affect GB transcriptional programs, highlighting the significance of organoid differentiation in modeling tumor–brain interactions ex vivo. Finally, we show that different GBs display distinct invasion patterns in rBOs that mirror in vivo growth, supporting the use of this ex vivo avatar system in therapeutic studies.

## Material and Methods

### Ethical

The Norwegian Food and Safety Authority granted approvals for establishing rBOs and orthotopic xenografts (approval numbers 19460 and 18611). Patient material was obtained from surgeries (Haukeland University Hospital) with written consent and approval from the Regional Ethics Committee (project numbers 013.09, 2020/65185, and 151825).

### Cell Culture

Five patient-derived GB cell lines (P3, BG5, BG7, GG6, and GG16) were derived from IDH1wt GB biopsies and grown in supplemented Neurobasal Medium as described previously.^13^ The H1-DL2 and H2-PIGF1 V600E BRAF 3D melanoma brain metastatic cell lines^14^ were grown in high-glucose Dulbecco’s modified Eagle’s medium as described previously.^15^

### Establishment of Brain Organoids

At E18, cortical regions from Sprague Dawley rat fetuses were dissected, dissociated, and 2.5 million viable cells were seeded into agar-coated T25 flasks. rBO development was monitored for 21 days under standard culture conditions. iPSC-derived BOs were generated using a published protocol,^3^ with added microfibers to increase surface-to-volume ratio.

### Immunohistochemistry and Immunofluorescence Staining

The BOs were fixed, embedded in paraffin, and sectioned using standard protocols for immunohistochemical analyses. Frozen sections were also prepared for immunofluorescence staining.

### Transmission Electron Microscopy

Twenty-one-day-old rBOs were prepared for electron microscopy according to standard procedures.^2^

### Transcriptome Analyses

At different rBO maturation times, RNA sequencing was applied using the Illumina TruSeq Stranded Total RNA preparation protocol. Ultra-low-input RNA sequencing, using the SMART-Seq v4 Ultra Low Input RNA Kit (TaKaRa), was employed to analyze the transcriptomes of flow-sorted invasive and noninvasive tumor cells. snRNA-seq was performed on a BGI DNBelab C4 system using droplet barcoding technology. All data were analyzed using standardized bioinformatics pipelines.

### Proteomic Analysis

rBOs as well as human iPSC BOs, were obtained at different stages of development. LC-MS/MS analysis was performed using a Q Exactive HF and Q Exactive Plus mass spectrometers (Thermo Scientific). Raw MS files were analyzed by MaxQuant version 1.6.2.3 (rBOs) and version 1.6.5.0 (iPSC BOs). For quantification and statistical analyses, label-free quantification (LFQ) was used.^16^

### Metabolomics Analysis

The extraction protocol of neurotransmitters was based on a protocol from Jäger et al.^17^ and analyzed by gas chromatography-mass spectrometry. Metabolite derivatization was performed using a multi-purpose sampler (GERSTEL).

### Animal Experiments and Magnetic Resonance Imaging

Orthotopic xenografts were established from P3, BG5, BG7, GG6, and GG16 tumors, as previously described.^18^ Tumor growth was monitored using a Bruker 7T Pharmascan MRI system (Bruker).^19^ The animals were sacrificed and perfused before CNV analysis and immunohistological examination.

### Cellular Barcoding

BG7 cells were labeled by lentiviral transfection rLV.EF1. Zs Green 1-9, rLV.EF1. AMCyan 1-9 and rLV.EF1. td Tomato 1-9 (Vectalys). Fresh NBM medium was changed after 72 h, at which point the barcoded cells were cultured for an additional week. Flow cytometry (BD FACSAria IIu Cell Sorter) was used to sort positive transfected cells.

### Co-Culture Experiments and Image Analysis

GFP-labeled (P3, BG5, BG7, GG6, GG16) and barcoded labeled tumor spheres were confronted with rBOs in 96-low attachment wells, whereupon invasion was assessed. For treatment, 10 ng/ml TGFβ (PeproTech), 10 µM TGFβ inhibitor (LY2157299, PeproTech), or 30 µM perampanel (Ad00Q) were added to the co-culture medium.

### GB Organoid Microtube Quantification

MT quantification was performed on confocal images of invasive regions in 4 eGFP-transduced GB models (BG7, P3, GG16, GG6) at the 72-hour time point. MTs were manually counted in 3 z-stacks of identical image size and visualized using ImageJ’s maximum intensity projection. Due to the high grade of invasion of BG5 at the given time point, this cell line had to be excluded from the analysis.

### Invasion Quantification

We developed a semi-automated method to quantify GB invasion, measuring (i) the competitive invasion ratio (core tumor cells replacing BO cells), (ii) the single-cell invasion ratio (tumor cells detached from the core), and (iii) total invasion as their sum.

### Statistical Analyses

Statistical analyses were performed using Student’s *t*-test, ordinary ANOVA or 2-way ANOVA together with Tukey’s multiple comparisons test. Analyses were performed using the GraphPad Prism Software (GraphPad). *P*-values for statistical significance were: *P < .05; **P < .01; ***P < .001; ****P < .0001; and NS, not significant.

## Results

### rBO Differentiation Follows a Developmental Path to Maturity That Reflects the In Vivo Brain Development

**Figure 1.**
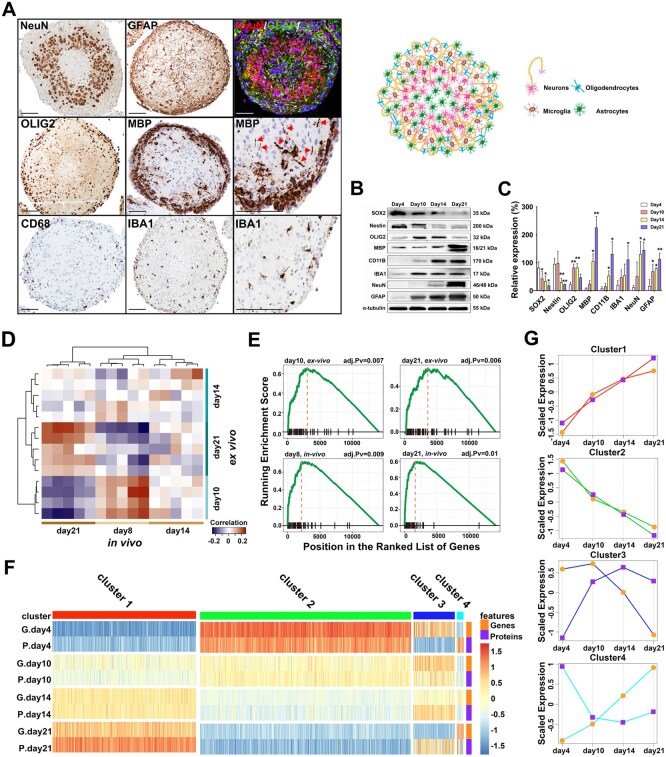
rBOs follow a strict developmental path to maturity. (A) Left panel: Immunohistochemical and immunofluorescence staining of neural cell markers in fully differentiated 21-day-old rBOs. Postmitotic neurons, detected by the neuron-specific nuclear protein NeuN. Glial fibrillary acidic protein (GFAP) shows a more even distribution of astrocytes within the rBOs whereas oligodendrocytes (OLIG2) positive cells are mainly localized in the rBO periphery where also myelinization takes place, as indicated by immunohistochemical staining for myelin basic protein (MBP; red arrowheads point at myelinated axons). Presence of microglia (IBA1) and macrophages (CD68) subpopulations within the rBOs. (Scale bar = 100 µm). Right upper panel: Schematic visualization of the cellular organization within the BOs based on the immunohistochemical data. See also Supplementary Figure S2A for further immunohistochemical stainings during the rBO development from day 4 to day 21. (B) Western blots of early CNS developmental markers (SOX2 and Nestin) show a gradual decrease during rBO differentiation, whereas markers of differentiation increase during rBO development: OLIG2: oligodendrocytes, MBP: myelin basic protein: CD11B: microglia/macrophages, IBA1: microglia, NeuN: neurons, GFAP: astrocytes. Shown is one representative image of the loading control α-tubulin. (C) Western blot quantification of markers. Data were generated from 3 independent organoid batches, *n* = 3. *P < .05, **P < .01 (ordinary one-way ANOVA and Bonferroni’s multiple comparisons test, mean ± SD). (D) A correlation matrix showing Spearman correlation coefficients calculated for the expression profiles of protein-coding genes measured following in vivo (*x*-axis) and ex vivo (*y*-axis) studies. (*n* = 4, each sample containing 10-20 rBOs). The dendrograms from hierarchical clustering are shown above and on the left side of the matrix. Red and blue colors correspond to positive and negative correlation coefficients, respectively. The time points are depicted below and on the right side of the plot. (E) Gene Set Enrichment Analysis plots. All 4 plots show enrichment of neuronal markers among the highly expressed genes at selected time points from the ex vivo and in vivo studies. The study type and the time points are shown above the corresponding plots. The *P*-values were adjusted using the Bonferroni–Hochberg method. (F) Transcriptomic (G) and proteomic (P) data show similar expression (clusters 1 and 2) within the rBOs at different developmental stages (days 4, 10, 14, and 21). The proteomic and transcriptomic data were linked based on gene symbol annotation. Four gene and protein clusters were identified (1-4). rBO sample size of days 4, 10, and 14: *n* = 5, rBOS sample size of day 21: *n* = 4. One sample consisted of 10-20 organoids. (G) Clusters 1 and 2 revealed a corresponding up- (cluster 1) or downregulation (cluster 2) during differentiation from 4 to 21 days. For cluster 3, RNA expression was high at 4 and 10 days with a sharp decrease at days 14 and 21 while protein expression increased during differentiation. An opposite phenomenon was observed for cluster 4.

rBOs were collected after 4, 10, 14, and 21 days of differentiation. Immunohistochemistry and regular staining revealed a cellular reassembly into highly organized structures on day 21, showing neurons with myelinated axons projecting from organized neuronal somas toward the rBO periphery (Figure 1A, Supplementary Figure S2A). Here, an abundant number of oligodendrocytes were also present. In contrast, astrocytes and microglia were scattered throughout the rBOs.

Moreover, quantitative immunohistochemical assessments showed a decrease and an increase in differentiation markers and a redistribution of cells toward rBO maturation (Supplementary Figure S2A and B). Cell proliferation (Ki67 expression) was also significantly reduced between days 4 and 21 (Supplementary Figure S2B). Electron microscopy revealed numerous synapses and myelinated axons within the rBOs (Supplementary Figure S2C).

Corroborating these findings, western blots revealed a consistent reduction of the stem cell markers SOX2 and Nestin from pnd day 4 to 21, whereas the oligodendrocyte marker OLIG2 increased from days 4 to 10 and 14, but then showed a slight decrease at day 21. Myelin basic protein (MBP) was strongly increased between days 14 and 21. The monocyte/macrophage marker (CD11B) showed a gradual increase from day 4 to 21, whereas the microglia marker (AIF-1/IBA1) showed a strong increase between day 4 and 10 followed by a consistent later expression. The neuronal marker (NeuN), expressed by both postmitotic excitatory and inhibitory neurons in the cortex, was strongly upregulated between days 14 and 21, whereas the astroglia marker (GFAP) showed a gradually increased expression during the differentiation period (Figure 1B and C).

To determine if rBOs at day 21 reached a high degree of differentiation, we performed RNA-seq on days 4, 10, 14, and 21 and compared them to in vivo rat brains (pnd 8, 14, and 21). Transcriptomics revealed a strong correlation between 10-day-old rBOs and pnd 8 brains, and between 21-day-old rBOs and pnd 21 brains, but with no correlation at day 14 (Figure 1D). Gene enrichment analysis confirmed increased neuronal marker expression in both the rBOs and the in vivo samples (Figure 1E).

To further delineate the developmental path to maturity, the rBO RNA-seq data were compared to results obtained from label-free LC‑MS/MS proteomic analysis at the developmental stages.

**Figure 2.**
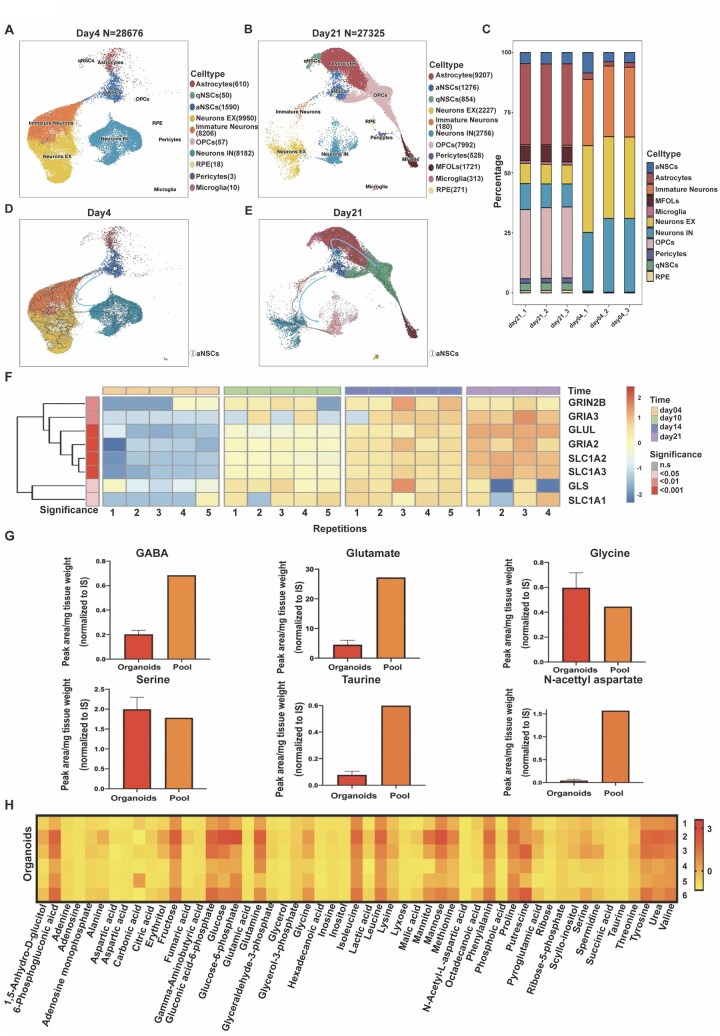
rBOs display cell-lineage differentiation over time and express central neurotransmitters. (A) Single-cell UMAP plot of day 4-rBOs shows 10 neural cell populations, where the most abundant are immature, excitatory (EX) and inhibitory neurons (IN). In addition, activated and quiescent neural stem cells (aNSC and qNSC) are observed, along with a smaller proportion of astrocytes and oligodendrocyte precursor cells. The high abundance of neuronal populations reflects what is seen in the postnatal brain in vivo. (B) Single-cell UMAP plot of day 21-rBOs shows 11 neural populations, where the most abundant are astrocytes, OPCs, and myelinating oligodendrocytes (MFOLs). Compared to 4-day-old rBOs, a significant shift has occurred toward gliogenesis, which is balanced by a decrease in the 3 neuronal populations. At this stage, microglia and pericytes are also seen. This figure clearly shows a differentiation towards maturity. (C) Quantitative assessment of the various neural populations within the rBOs. (D) Pseudotime developmental trajectories in day 4-rBOs are evident from aNSC to immature neurons, and then to excitatory and inhibitory neurons. These trajectories reflect the early stages of brain development. A limited developmental process is also seen from aNSCs towards astrocytes (depicted by arrows). Analysis was done using Monocle3 (spatial correlation measured by Moran’s *I* statistic). (E) Pseudotime developmental trajectories in day 21 rBOs are clearly directed toward gliogenesis: Astrocyte-OPC-and MFOLs (depicted by arrows). Note the virtual absence of immature neurons and the reduction of aNSCs, indicating a highly differentiated state. Analysis was done using Monocle3 (spatial correlation measured by Moran’s *I* statistic). (F) Heat map generated from RNA-seq data focusing on ionotropic glutamate receptors involved in fast synaptic transmission (AMPA receptors; GRIA2&3), NMDA receptors (GRIN2B), as well as genes involved in the Glutamate/GABA–Glutamine cycle (GLS, GLUL, SLC1A, SLC2, and SLC1A3) during rBO differentiation (day 4 to 21). (G) Comparison of central neurotransmitter expression between rBOs and normal rat brain tissue (Pool: *n* = 4). In general, intermediates of central carbon metabolism (including the tricarboxylic acid cycle associated with amino acids aspartate and glutamate) and polyamines were lower compared to normal brain tissue. Both glutamate and GABA were detected in the rBOs, also at lower levels compared to normal brain tissue. Interestingly, serine and glycine, known to be important in the regulation of excitatory glutamatergic transmission, are highly expressed in the rBOs. (H) Metabolome mass spectrometry heatmap on 21-day-old rBOs (*n* = 6) showing branched-chain amino acids like isoleucine, leucine, and valine to be upregulated in the organoids compared to normal rat brain tissue. These are known to have an important role in the synthesis of excitatory glutamate as well as in the synthesis of the inhibitory GABA neurotransmitters.

Principal component analyses (Supplementary Figure S3A and B) and hierarchical clustering revealed increasing differences in the global proteome of the rBOs across different developmental stages, with the highest number of differential proteins observed between day 4 and day 21 rBOs. The RNA-seq data, to a large extent, reflected the proteomics data according to their developmental stage (Supplementary Figure S3C-F). After proteomic analysis, we investigated whether changes in the proteome during differentiation aligned with transcriptomic data. Proteomic and transcriptomic data were linked by gene symbol annotation, identifying 1320 common genes and proteins with FDR < 0.01 (Figure 1F). Hierarchical clustering revealed 4 clusters (1-4). Clusters 1 and 2 showed corresponding up- (cluster 1) or downregulation (cluster 2) at both RNA and protein levels during differentiation from 4 to 21 days. In cluster 3, RNA expression decreased from day 14, while protein levels increased, with the opposite trend observed in cluster 4 (Figure 1G). The gene ontology of cluster 1 showed upregulation in neurotransmitter processes, while cluster 2 involved downregulation of early organ development processes (Supplementary Figure S4A). Moreover, a deep proteomic analysis of the BOs (cultured for 4, 10, and 21 days) allowed us to further compare numerous markers associated with CNS development. Cell proliferation (Ki67) and stem cell markers (Musashi-1; MSI1) and Nestin were significantly reduced from day 4 to day 21 (Supplementary Figure S4B). In contrast, the mature neuronal marker SYPH and the astrocyte marker GFAP, as well as the oligodendrocyte differentiation markers MBP and PLP1 were increased (Supplementary Figure S4C). The GABA transporters SLC6A1 and SLC6A11, known to be involved in the rapid removal of GABA to maintain low extracellular levels, were increased between 4 and 21 days. Also, the glutamate receptor proteins GRIN2B and GRID1 showed a strong increase during the same period (Supplementary Figure S4D). To further corroborate these findings, we performed single-cell nuclear RNA sequencing (scRNA-seq) comparing 4-day-old rBOs to 21-day-old rBOs. These results revealed a remarkable cellular lineage differentiation from day 4 to day 21-old rBOs. At day 4 (Figure 2A), 10 neural populations were identified with the most abundant being 3 neuronal populations. A high number of both excitatory (EX) and inhibitory (IN) neurons were present, as seen in the postnatal brain in vivo,^20^ as well as a significant proportion of immature neurons. In addition, there were small proportions of quiescent (q) and activated (a) neural stem cells (NSC), as well as a small proportion of astrocytes and oligodendrocyte precursor cells (OPC). Very few microglia and pericytes were seen. Thus, the 4-day-old rBOs exhibit a similar cellular composition as seen in the postnatal brain in vivo. At day 21 (Figure 2B), 11 neural populations were identified, where the most abundant were astrocytes, OPCs, and myelinating oligodendrocytes, indicating extensive gliogenesis. Compared to 4-day-old rBOs, a marked decrease was observed in the 3 neuronal populations, which most likely is caused by the establishment of a strict cellular equilibrium between neurons and glia cells within the rBOs. At this stage, microglia and pericytes were also observed. This figure clearly demonstrates a development trajectory towards a mature state. The quantification of the various cell populations is depicted in Figure 2C.

Moreover, following a pseudotime trajectory analysis with activated multipotent, neural stem cells (aNCS) as a starting point, we show a clear developmental trajectory:


aNSC→Immature neurons→EX neurons→IN neurons

This path reflects early brain development, where EX neurons are formed earlier than IN neurons. From the aNSCs there is also a small branch directed towards astrocytes (Figure 2D, Supplementary Figure S4E).

At day 21, most of the developmental trajectories were toward gliogenesis, mainly directed towards astrocytes, OPCs, and MFOLs (Figure 2E, Supplementary Figure S4E). This clearly demonstrates that the rBOs develop along biologically meaningful developmental paths, ultimately reaching a point of equilibrium where gliogenesis dominates and neuronal numbers stabilize.

As shown in Supplementary Figure S2C, rBOs contained numerous synapses. The observed developmental trajectories suggest that this maturation requires neuronal and metabolic connectivity, which is further supported by Gene Ontology analysis of the single-cell data (Supplementary Figure S4F). Taken together, these results indicate that the rBOs reach a high degree of differentiation by postnatal day 21.

### rBOs Express Transmitters Involved in Neuronal Function

We next analyzed the transcriptomic datasets at different time points of differentiation (days 4, 10, 14, and 21) and for snRNA-seq (days 4 and 21) based on a 50-gene signature (Supplementary Figures S5 and S6), focusing on major genes involved in glutamate turnover, as well as the corresponding glutamate receptors (AMPA and NMDA). Specifically, we examined the expression of regulatory genes involved in the glutamate/GABA–glutamine cycle, a pathway essential for neurotransmitter balance. Neurons release GABA, which astrocytes take up and convert into glutamine, later used by neurons to synthesize glutamate or GABA. During differentiation (Figure 2F), AMPA (GRIA2,3) and NMDA (GRIN2B) were significantly upregulated, along with glutamate transporters (SLC1A, SLC2, SLC1A3), glutaminase (GLS), glutamine synthetase (GLUL), glutamate dehydrogenase (GLUD1), and GABA transaminase (ABAT), which regulates GABA turnover.

We subsequently performed metabolome mass spectrometry on 21-day-old rBOs and pooled normal rat brain tissue (Figure 2G). Intermediates of central carbon metabolism (eg the tricarboxylic acid cycle involving aspartate and glutamate) and polyamines were lower compared to brain tissue (data not shown). This difference may result from rBOs being cultured in nutrient-rich media, unlike the brain tissue, which has very different metabolite concentrations within its microenvironment. Despite this, using a targeted chromatographic technique,^17^ we detected 6 out of 14 neurotransmitter-related metabolites in the rBOs (Figure 2G).

Levels of histamine, serotonin, epinephrine, and norepinephrine were too low in abundance and could not be detected in either the rBOs or the brain samples. Both glutamate and GABA were detected in the rBOs, although at lower levels compared to normal brain tissue. Interestingly, serine and glycine were found to be highly abundant in the rBOs. These are known to be important in the regulation of excitatory glutamatergic transmission, where they act as co-agonists of the NMDA subtype of glutamate receptor.^21^ Consistent with these observations, the snRNA-seq data showed, through GO enrichment analysis, a marked upregulation of functions related to CNS metabolism and neural connectivity (Supplementary Figure S4F).

The analysis of the rBOs further revealed that several amino acids, including branched-chain amino acids (isoleucine, leucine, and valine), were upregulated, which are involved in synthesizing excitatory glutamate and inhibitory GABA transmitters (Figure 2H).^22^

### rBOs Show a Higher Degree of Differentiation Compared to BOs Derived from Human iPSCs

Using a highly standardized iPSC BO protocol, we confirmed that the cellular composition matched prior studies (Supplementary Figure S7A).^23^ LC-MS/MS analysis on 1, 3, and 4-months old iPSC BOs revealed 1652 differentially expressed proteins between the developmental stages (FDR < 0.01). Hierarchical clustering showed a clear separation between months 1, 3, and 4, with the largest difference between months 1 and 3 (Supplementary Figure S7B). Gene ontology analysis of proteins from months 1 and 4 highlighted processes like synaptic vesicle cycle, neuronal projections, and synaptic plasticity (Supplementary Figure S7C).

The detailed proteomic data from both rBOs at days 4, 10, 14, and 21, and from iPSC BOs at months 1, 3, and 4, allowed us to compare the levels of numerous markers associated with CNS development. As for the rBOs, the iPSCs BOs showed a significant decrease in the cell proliferation marker Ki67 and the stem cell marker Musashi-1 (MSI1) (Supplementary Figure 7D). Nestin expression was reduced in the rBOs but not in the iPSC BOs (Supplementary Figures S4B and S5D). For both models, an increase was seen in the mature neuronal marker SYPH and the astrocyte marker GFAP (Supplementary Figures S4C and S7D). The GABA transporters SLC6A1 and SLC6A11, as well as GRIN2B, GRID1, MBP, MAG, and PLP, were not detected in the iPSC BOs.

We next correlated the rBOs’ and iPSC BOs’ protein profiles at different developmental stages. As shown in Supplementary Figure S7E, the protein expression profiles from 4-month-old iPSC BOs corresponded more to rBOs at day 10, whereas the highest correlation of 1-month-old iPSC BOs corresponded to 4-day-old rBOs, and more likely, with an even earlier developmental stage.

In conclusion, the presented data show that rBOs at day 21 display a higher degree of brain maturation compared to iPSC-BOs.

### Different Human GBs Show Variations in Invasion Patterns in rBOs That Reflect Tumor Growth In Vivo

**Figure 3.**
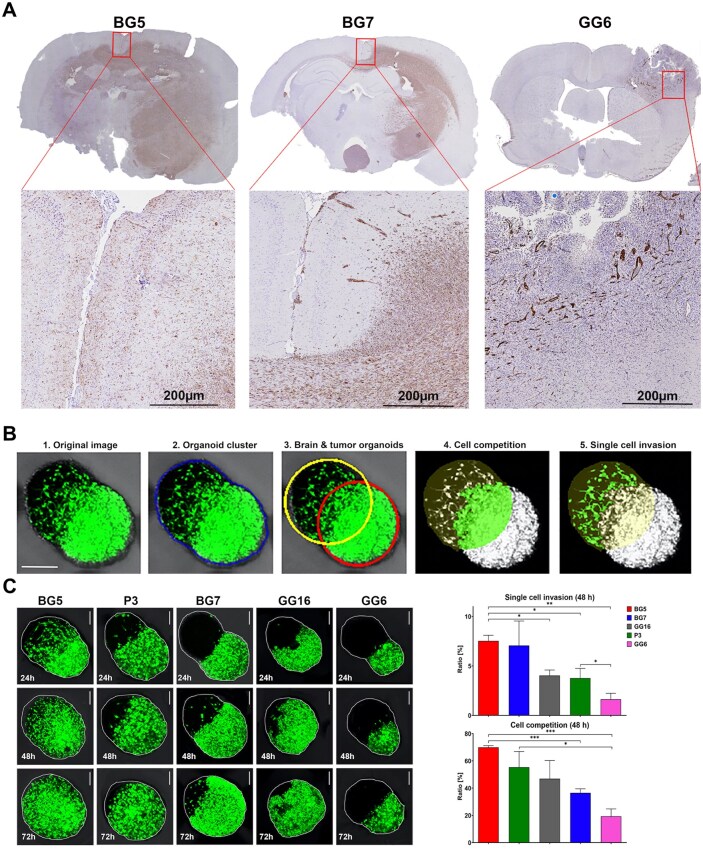
Tumor cell invasion in vivo is reflected in cocultures in vitro. (A) Detailed immunohistochemical analyses, using an anti-human Nestin antibody, show an extreme infiltrative growth of BG5 tumors where tumor cells are present in most brain compartments. BG7 shows an extensive invasion along the corpus callosum as well as perivascular infiltration. GG6 displays extensive angiogenesis, pseudopalisading necroses with limited single-cell infiltration into the brain parenchyma. Interestingly, the main tumor mass of GG6 was Nestin negative, yet with positive cells around blood vessels. P3 and GG16 exhibit the same invasion patterns as BG7 and have been previously published elsewhere.^30^^,^^43^ (B) Example of quantification of invasion of the BG5 tumor after 24 h of confrontation with a rBO. 1: Original image of merging organoids. 2: The contour of the organoids cluster is obtained using a region-growing algorithm with a seed point inside the cluster. 3: Yellow (brain) and red (tumor) ellipsoids are drawn whose curvature follows the periphery of the merging organoids. 4: Cell competition represents the tumor mass (GFP-positive pixels) that has replaced the brain tissue within the rBO. (C) Left panel: Z-stack confocal images, showing different invasion patterns of 5 different GFP-labelled GB organoids (BG5, P3, BG7, GG16, and GG6) into the rBOs. Images were obtained after 24, 48, and 72 h following tumor confrontation with the rBOs. The invasion patterns mirror their mode of invasion in vivo (Figure 3A). Scale bar = 100 µm. Right panel: Two invasion parameters were assessed by the automated quantification analysis using metrics for brain invasion. The upper panel depicts the ratio of single-cell invasion to rBO area for the different GBs, whereas the lower panel shows the amount of rBOs that have been out-competed (replaced) by the main tumor mass. Measurements were obtained at a 48-h time point. Data from 3 individual co-culture experiments are presented as mean ± SEM. (*P < .05, **P < .01, ***P < .001, *n* = 3, unpaired *t*-test).

We first assessed invasion in vivo using 5 isocitrate dehydrogenase (*IDH*) wild-type GBs patient-derived xenotransplantation models (P3, BG5, BG7, GG6, and GG16), where tumor growth was assessed by high-resolution 7T MRI (Supplementary Figure S8A and B). Detailed histological analyses revealed that the BG5 tumors were characterized by an extreme infiltrative growth with single cells present in most brain compartments. (Figure 3A, Supplementary Figure S8B). BG7, P3, and GG16 displayed extensive invasion along the corpus callosum as well as perivascular infiltration but with a more collective invasion pattern compared to BG5 (Figure 3A, Supplementary Figure S8B). GG6 displayed extensive angiogenesis with limited single-cell infiltration into the brain parenchyma (Figure 3A).

We then developed an automated method to quantify invasion by measuring brain invasion (the brain organoid area invaded by tumor cells) and the invasive fraction (the ratio of invasive to total tumor cells). This enabled us to measure single-cell invasion into the rBOs and cell competition (ie, the amount of brain tissue replaced by the main tumor mass) (Figure 3B). Based on in vivo invasion patterns, we co-cultured GFP-expressing tumors (P3, BG5, BG7, GG16, and GG6) with mature rBOs and assessed tumor cell invasion at different time points (24, 48, and 72 hours) using confocal microscopy. BG5, BG7, P3, and GG16 all showed individual tumor cell invasion into the rBOs, while GG6 exhibited limited invasion (Figure 3C, Supplementary Movie I). The automated quantification at 48 hours (Figure 3C) reflected the invasion patterns observed in vivo, with extensive single-cell invasion for BG5 and limited invasion for GG6. Notably, single-cell invasion was clearly observable and quantifiable at 24 and 48 hours. By 72 hours, however, cell competition predominated, as major parts of the rBO had been replaced by the main tumor mass.

Brain metastases from secondary tumors typically grow as well-defined lesions within the brain.^24^ As negative controls, we used 2 well-characterized human brain melanoma metastasis models (H1-DL2 and H2-PIGF1) that metastasize to the brain after injection into the left cardiac ventricle.^14^

Tumor spheroids from these cell lines strongly attached to the rBOs but showed no invasion into the rBOs, as also reflected by their growth in vivo (Supplementary Figure S9A and B).

In conclusion, tumor spheres derived from different human GBs show different rBO invasion patterns, which are reflected by their growth in vivo. Moreover, the invasion into the rBOs represents a glioma-specific phenomenon not reflected by secondary brain metastases.

### The rBO Microenvironment Induces Profound Transcriptional Changes in the GB Cells

**Figure 4.**
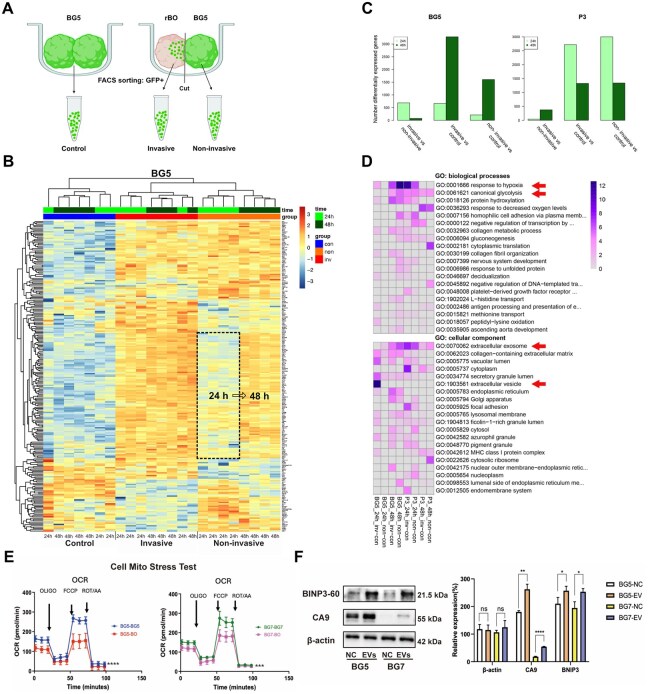
Transcriptomic analyses of GB invasion into rBO. (A) Schematic presentation of isolation of invasive and noninvasive GB cells for transcriptome analysis. GFP-positive BG5 and P3 tumor spheres were confronted with mature rBOs for 24 and 48 h. As controls, we used the confrontation of 2 tumor spheres. After 24 and 48 h, cocultures were cut in half, and GFP-positive cells were isolated using dissociation and flow cytometric sorting. This allowed us to isolate the invasive cells within the rBO part (invasive tumor cells) and the tumor mass next to the organoid (noninvasive compartment). As a control, GB tumor sphere-co-cultures were confronted for 24 and 48 h, dissected, and sorted in the same way. (B) Heatmap of top differentially expressed genes, in BG5 cocultures, across control, invasive, and noninvasive compartments using unsupervised clustering. A distinct separation of control, invasive, and noninvasive cells is seen. Rows represent individual genes, and columns represent individual samples (replicas) at various time points (24 h, 48 h). Both the invasive and noninvasive compartments show marked differences in gene expression (up- and downregulated genes) after confrontation with rBOs. The highlighted square emphasizes a subset of genes that are dynamically changed in the noninvasive compartment between 24 and 48 h, upregulated in invasive tumor cells at 24 h and subsequently in noninvasive tumor cells suggesting a time-dependent activation of this gene cluster following confrontation with the rBOs. (C) Bar plot illustrating the number of differentially expressed genes (DEGs) between invasive and noninvasive cells, invasive and control, and noninvasive and control at 24 and 48 h for BG5 and P3. In P3, the highest number of DEGs is observed at 24 h after confrontation with rBOs, indicating significant early changes in gene expression with minimal differences between invasive and noninvasive cells. In contrast, BG5 shows an initial increase in DEGs in invasive cells at 24 h. The main changes occur at 48 h, with a remarkable increase in DEGs in both the invasive and noninvasive cells compared to control. This indicates a delayed, yet substantial, gene expression response in BG5, with early changes in invasive cells followed by significant changes in both invasive and noninvasive cells at 48 h. (D) Functional annotation of significantly upregulated genes in invasive and noninvasive BG5 and P3 cells compared to controls, showing similar enrichment in gene ontology (GO) terms after confrontation with rBO. Both BG5 and P3 tumor cells show enrichment of genes associated with response to hypoxia and glycolysis as well as extracellular exosome activity. (E) Assessment of mitochondrial respiration (OCR) using a Seahorse XF Flex Analyzer of BG5- (red) and BG7-rBO (magenta) cocultures. As control, we used tumor-tumor spheroid confrontations (BG5-BG5 in blue and BG7-BG7 in green). The tumor-rBO cocultures have lower OCR than the corresponding tumor-tumor co-cultured cells (BG5-BG5 (blue) and BG7-BG7 (green)). This strongly suggests impaired oxidative phosphorylation and a compensatory increase in glycolysis. Immunostaining for 2 hypoxia markers CA9 and BNIP3 after exposing BG5 and BG7 with EVs harvested from 21-day-old rBOs revealed an upregulation in the tumors following rBO-EV exposure.

To identify transcriptional programs involved in GB invasion, we extracted single tumor cells invading the rBOs for gene expression analysis. The rBOs were confronted with BG5 and P3 tumors for 24 and 48 hours. To isolate invading GFP-labeled tumor cells, cocultures were cut in half under a dissecting microscope (Figure 4A). After dissociation and flow cytometric sorting, ui-RNA-seq was performed. Controls included BG5 and P3 cocultures not exposed to rBOs. In BG5-rBO cocultures, unsupervised clustering revealed distinct gene expression differences between invasive and noninvasive compartments after confrontation. The highlighted square shows a subset of genes that are dynamically changed in the noninvasive compartment between 24 and 48 hours (Figure 4B). These genes were upregulated in invasive tumor cells at 24 hours and later in noninvasive cells, suggesting a time-dependent GB cell activation after rBO confrontation. Figure 4C shows the number of significantly differentially expressed genes (DEGs) between invasive, noninvasive, and control cells at 24 and 48 hours for BG5 and P3. In P3, DEGs peaked at 24 hours, indicating early gene expression changes after confrontation, while BG5 showed an initial change in DEGs in invasive cells at 24 hours, followed by major changes in gene expression occurring at 48 hours in both cell compartments. This suggests a delayed yet strong transcriptional response in BG5, while P3 exhibits earlier changes.

Overall, gene ontology analysis revealed that the rBOs induce significant gene expression shifts in both models towards hypoxia, canonical glycolysis, and protein hydroxylation, indicating that the rBOs reprogram the tumor cells toward glycolysis (Figure 4D). The analysis further indicates that this reprogramming occurs through the release of extracellular vesicles (EVs). These findings align with prior studies indicating that extracellular exosomes may play a crucial role in tumor–host communication by facilitating tumor cell invasion.^25^ To validate these findings, we first assessed mitochondrial respiration (OCR) using a Seahorse XF Flex Analyzer. As seen in Figure 4E, both BG5-rBO and BG7-rBO cocultures had a significantly lower OCR than BG5-BG5 and BG7-BG7 co-cultured cells. This strongly suggests impaired oxidative phosphorylation and a compensatory increase in glycolysis. Secondly, our bioinformatic analyses also suggested that rBO reprogramming occurs through the release of extracellular EVs. We therefore harvested EVs from 21-day-old rBOs and exposed them to BG5 and BG7 (Supplementary Figure S8). Following co-incubation with the EVs, we performed immunostaining for CA9 and BNIP3, both excellent markers of hypoxia. As shown by the western blots (Figure 4F), CA9 and BNIP3 were strongly up-regulated in the tumors following rBO-EV exposure.

In conclusion, we demonstrate that the rBOs dynamically reprogram the metabolism of tumor cells to favor hypoxic pathways, thereby enhancing invasion. A further mechanistic insight into these highly interesting results warrants further investigation.

### The rBO Differentiation Status Influences GB Invasion

Since immature rBOs have different transcriptomic and proteomic profiles compared to mature rBOs (Figures 1 and 2), we investigated whether the rBO differentiation status affected the invasive process. Surprisingly, invasion was significantly reduced in 4-day-old rBOs compared to differentiated rBOs despite their looser brain structure (Supplementary Figure S11). This suggests that the BO maturation status is important when assessing GB invasion ex vivo. More research is required to determine whether these results apply to human BOs.

### Multiple GB Cell Clones Show the Ability to Invade the rBOs, Where Their Invasion Patterns and Speed Can be Measured in Real-Time

**Figure 5.**
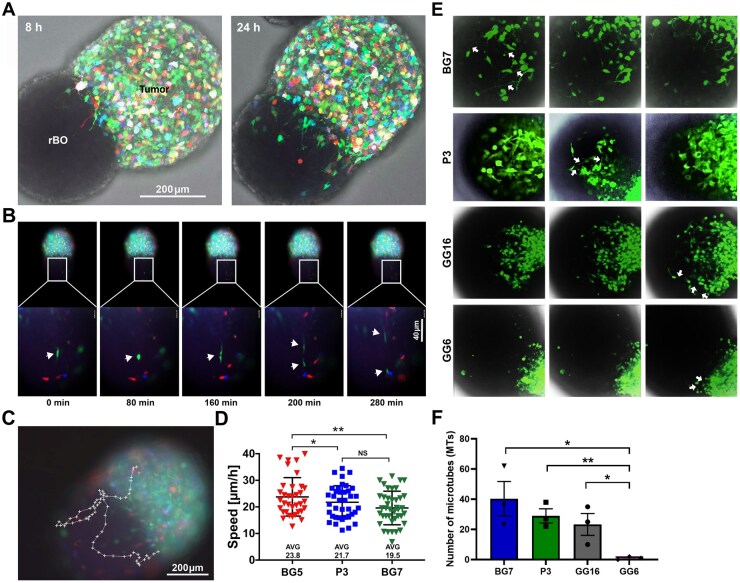
Image analysis of GB invasion into rBOs. (A) A barcoded GB organoid (BG7) confronted with a rBO visualized at 8 and 24 h of confrontation. After 8 h, fingerlike protrusions can be seen projecting into the rBO. At 24 h multiple clones are seen invading into the rBO. (B) Images obtained from the time-lapse movie (Supplementary Movie II) show a tumor cell undergoing mitosis over a ∼160 min time period within the rBO. Immediately following cell division, the 2 daughter cells migrate in opposite directions. (C) A typical tracking pattern of a tumor cell within the rBO. When the tumor cells reached the counter-lateral margin of the rBO, they turned around and moved back towards the tumor organoid. (D) The invasion speed of different cell clones within the rBO was assessed by tracking cell invasion trajectories (Supplementary Movie III). BG5 tumor cells exhibited a significantly higher invasion speed compared to P3 and BG7. The invasion speed of individual cell clones within the rBO was tracked using video recordings taken every 24 h for 40 min of each coculture with P3, BG5, and BG7. The total observation period was 72 h. Each data point represents the distance traveled within 40 min video recording. The covered distance was measured using the Imaris Bitplane imaging software and converted to speed (µm/h). Data are represented as individual data points, mean (horizontal line), and ± SD. (Analyses were done on 35 to 40 individual cell movements, *P < .05, **P < .01, one-way ANOVA with Student–Newman–Keuls (SNK) post hoc test). (E and F) Quantification of tumor microtube (TM) networks within the cocultures. GB organoid microtube formation and quantification using 4 different eGFP-transduced GB models (BG7, P3, GG16, and GG6). (E) z-stacks using the maximum intensity projection (MIP) tool of ImageJ. White arrows indicate examples of MTs, showing how the quantification was performed. (F) Quantification data showing a significantly higher amount of MTs in BG7, P3, and GG16 compared to GG6 (unpaired *t*-test, *P* < .05). Data are presented as mean ± SEM.

We and others have recently shown that GB organoids, as well as their orthotopic xenografts, represent clinically relevant GB models.^7^^,^^18^ To determine if invasion into the rBOs was attributed to a specific GB clonal population, we barcoded them with specific fluorescent markers (GFP, RFP, and Cyan) (Supplementary Figure S12A). The barcoded GBs were then confronted with the rBOs, and shortly after the confrontation, fingerlike protrusions from the GB cells were seen projecting into the organoids (Figure 5A). Time-lapse fluorescence imaging revealed that multiple clones showed the ability to invade the rBOs, indicating that cell invasion is not attributed to a specific clonal population (Figure 5A, Supplementary Movie II).

The “Go or Grow” hypothesis, which has been debated, proposes that tumor cells delay cell division to facilitate migration.^26^ By following the barcoded invasive cells within the rBOs, we observed sudden events where the cells divided, whereupon they immediately continued their invasion (Figure 5B, Supplementary Movie II). These experiments demonstrate a putative “Go and Grow” behavioral pattern of single cells within the invasive tumor/brain compartment. Recent studies have supported this notion.^27^

Using Imaris Bitplane imaging (https://imaris.oxinst.com), the invasive cells were tracked within the rBOs. Interestingly, the invasive cells did not migrate out of the organoids but rather changed their direction when they reached the rBO periphery (Figure 5C, Supplementary Movie II). Moreover, we were able to determine the invasion speed within the rBOs. BG5, P3, and BG7 showed an average speed of 23.8, 21.7, and 19.5 μm/h, respectively (Figure 5D, Supplementary Figure S12B, Supplementary Movie III).

In conclusion, we show that multiple GB clones invade into the rBOs as single independent cells with an invasion speed of 19-24 µm/h. Moreover, the invasive cells undergo cell divisions with a delay in invasion only during mitosis.

### The Amount of Tumor Microtubes Within the Cocultures Is Associated with Tumor Cell Invasiveness

Tumor microtubes (MT) formation and cellular interconnections have been shown to play a role in tumor cell invasion, proliferation, and radioresistance.^28^ Neuronal GB connections leading to MT formation have been shown to be associated with increased invasion speed.^29^ Also, TM-network unconnected cells can display invasive and proliferative capacities before they transition into an interconnected TM network.^29^

Given the putative importance of the TM network in GB invasion, we quantified the TM network in BG7, P3, GG16, and GG6 cocultures that show different invasive capacities (Figure 3). As demonstrated in Figure 5E and F, the number of TMs was significantly higher in BG7, P3, and GG16 compared to GG6, reflecting their invasive capacities. We conclude that TMs are formed in the cocultures, and an increased number of TMs is associated with enhanced invasive capabilities.

### The Utility of rBOs to Assess Therapeutic Interventions

**Figure 6.**
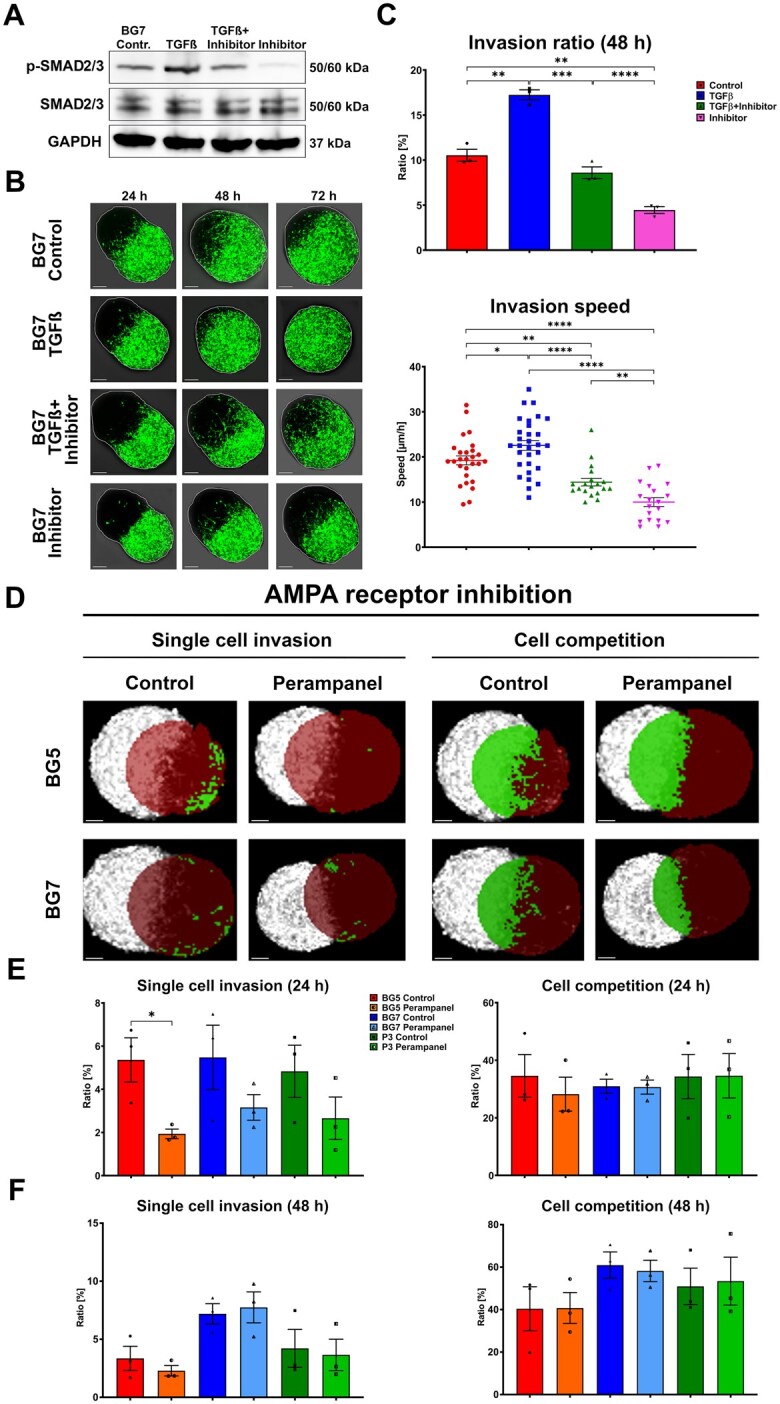
The cocultures can be used to assess therapeutic interventions. (A) Western blot analysis of BG7 tumor organoids shows a strong upregulation of p-SMAD2/3 after TGFβ treatment, while the TGFβ-specific inhibitor, LY2157299, downregulates p-SMAD2/3. (B) Z-stack confocal images of BG7-rBO cocultures after 24, 48, and 72 h of treatment with either TGFβ, the combination of TGFβ and LY2157299, or LY2157299 alone. The rBOs are fully invaded by tumor cells after 72 h of TGFβ treatment, whereas TGFβ inhibition led to the opposite effect. LY2157299 alone was not able to fully block the invasive process. (C) Quantification of invasion ratio after 48 h of treatment with TGFβ showing an increased invasion capacity and speed of BG7 tumor cells. A decline in invasiveness is detected following TGFβ inhibition. Data from 3 separate experiments are plotted as mean with SEM for the invasion ratio (upper panel). The invasion speed data are represented as individual data points, mean (horizontal line), and SEM (lower panel). For the calculation of the invasion speed, 19 to 32 individual cell movements were tracked. *P < .05; **P < .01, ***P < .001, ****P < .0001 (*n* = 3, unpaired *t*-test). (D) For BG5 tumors, 24 h treatment of cocultures with the AMPA glutamate receptor antagonist perampanel (30 µM) leads to a significant decrease of single-cell invasion into the rBOs, whereas no effect on cell competition was observed. Regarding single-cell invasion, a similar trend was shown for cocultures with BG7 and P3 organoids. The cell competition ratio is not reduced for perampanel-treated P3 and BG7 cocultures. The data are shown as mean with SEM from 3 individual experiments (*p < .05, unpaired *t*-test). (E) Following 24 h of perampanel treatment, a significant reduction was observed for the most invasive GB model (BG5) but with also a trend in P3 and BG7. (F) Following 48 h treatment a trend in reduced single-cell invasion was also observed for P3 and BG5, but not for BG7. For cell competition, only a slight invasion reduction was noticeable for BG5. Presented are mean ± SEM.

GB aberrant transforming growth factor beta (TGFβ) signaling has been shown to have multiple effects on cell migration and invasion.^30^^,^^31^ As a first proof of concept on how the invasion model can be used in a therapeutic context, we determined to what extent TGFβ stimulation, as well as a TGFβ-specific inhibitor, LY2157299, affected the invasive process. For this purpose, we performed western blots on BG7 3D tumors focusing on a key signal transducer involved in TGFß signaling; SMAD 2/3. As shown in Figure 6A, TGFß stimulation led to a strong upregulation of p-SMAD 2/3, whereas inhibition led to its downregulation. TGFβ, TGFβ + LY2157299 as well as LY2157299 alone were then added to the co-culture system, and the invasion process was quantified at 24, 48, and 72 hours. As shown in Figure 6B and C, TGFβ significantly enhanced the invasion capacity and increased the invasion speed of BG7 tumor cells, whereas TGFβ inhibition led to an opposite effect. Yet, TGFβ inhibition alone was not enough to fully block invasion.

It has recently been shown that GB cells can form synapses with neurons, pointing at an extensive communication network between brain cells and tumor cells.^32^ This network is, to a large extent, mediated via the metabolites glutamine and glutamate, both needed for GB cell proliferation. It has been shown in preclinical models that perampanel, an antiepileptic agent that functions as a specific noncompetitive AMPA glutamate receptor antagonist, has an inhibitory effect on GB growth.^33^ Since the rBOs possess the molecular machinery for AMPA and NMDA receptor assembly, including accessory proteins involved in the glutamate/GABA–glutamine cycle, we queried to what extent AMPA receptor inhibition affected the invasive process. For this purpose, we utilized the highly infiltrative BG5 model, which has been demonstrated to establish synapses with neurons in vivo,^33^ as well as with BG7 and P3 tumors. As shown in Figure 6D and E, perampanel treatment led to significant inhibition of the ratio of single-cell invasion of BG5 cells by 63% (*P *= .0306) after 24 hours of treatment. A similar trend was observed for BG7 and P3. After 48 hours, single-cell invasion inhibition was less evident, as seen by reduced cell competition (replacement of rBO by the main tumor mass) (Figure 6F). Notably, perampanel treatment had no significant effect on cell competition, suggesting that brain tissue replacement by the tumor is driven by other mechanisms. In conclusion, the rBO co-culture model described represents a promising tool for assessing new therapeutic strategies towards the glioma cell invasive compartments.

## Discussion

There are 2 primary methods for generating BOs. The first involves dissociating cells from developing brain tissue and reaggregating them into self-organizing 3D structures.^34^ The second involves the use of human pluripotent stem cells (hPSCs), which, through specific protocols, form BOs that recapitulate the cellular patterns and organization of the developing human brain.^3^ These BOs can be produced via an unguided approach, with minimal external inputs, or through a guided approach that involves adding small molecules, growth factors, or specific cell types to increase cellular complexity and differentiation.^35^^,^^36^

iPSC-derived cerebral organoids have been used as a scaffold to study human GB invasion parameters, revealing changes in tumor cell gene expression necessary for their spread. However, these interactions were studied using 17-day-old BOs,^8^ which, at this stage, represent highly immature developmental structures. Similarly, GB stem cells have been shown to invade human embryonic stem cell-derived BOs, forming tumors within them that phenocopied the patient-derived GBs.^37^ Our data indicate, however, that fully differentiated brain tissue may be essential for studying GB invasion ex vivo, since a significantly reduced GB invasion was observed in immature 4-day-old rBOs compared to 21-day-old rBOs (Supplementary Figure S11).

A key part of this work, therefore, focused on characterizing rBOs at the cellular and molecular levels. We have shown by snRNA-seq that the rBOs exhibit a remarkable cellular complexity with a clear neural cell lineage differentiation towards a highly differentiated state, leading to uniform structures containing microglia, astrocytes, oligo­dendrocytes, and myelinated axons (Figures 1 and 2). In this context, it is known that altricial rodents experience greater CNS immaturity but undergo rapid brain development compared to humans. Consequently, major developmental processes are compressed within a short neonatal period.^38^ At pnd 21, the rat brain reaches ∼95% of its adult weight and a peak in its myelinization rate as well as neurotransmitter and receptor changes,^25^ indicating terminal differentiation. A potential limitation of using rBOs compared to human BOs is that the co-culture system is heterogeneic. However, it has been shown that human BOs, when transplanted into rodent brains, integrate within the brain tissue, forming functional connections and supporting interspecies communication.^39^ A further limitation of rBOs, reflecting most BOs, is the absence of a vascular network. Our snRNA analysis detected a minor population of pericytes, whereas endothelial cells were not observed. The functional significance of the pericytes within rBOs remains to be determined. Several novel observations should be highlighted in the presented study. We demonstrate that GB cells invading rBOs display patterns similar to those observed in vivo (Figure 3), and that multiple clones exhibit invasive capacities (Figure 5A, Supplementary Movie II). We also show that individual cells can invade and divide independently, rather than as interconnected populations (Figure 5B). This partly challenges the “Go or Grow” hypothesis, which posits that glioma cells alternate between migration and proliferation to drive diffuse invasion and resistance to therapy.^26^^,^^40^ Our findings further highlight that single-cell invasion is a highly dynamic process, with individual GB cells exchanging positions within rBOs at variable speeds, depending on the tumor model (Figure 5C and D, Supplementary Figure S12). This dynamic behavior can be captured by quantifying 2 complementary parameters: single-cell invasion and cell competition. We further show that the rBOs, by the release of EVs, reprogram the tumor cells towards hypoxia. This mechanism may be important, as it has previously been demonstrated that hypoxia can promote GB invasion by activating HIF-dependent pathways that enhance motility and perivascular infiltration.^19^^,^^41^

Beyond its value as a biological model, the co-culture system also provides a platform for therapeutic exploration. As a proof of concept, we demonstrate that invasion dynamics can be modulated through TGFβ stimulation or inhibition, as well as by interfering with tumor-neuronal AMPA receptor synapses using perampanel. As expected, TGFß-stimulated invasion and its inhibition by LY2157299 reduced the invasive process. Yet, TGFß inhibition did not lead to complete inhibition of invasion (Figure 6). It has recently been shown by single-cell sequencing that invasive glioma cells display an increased expression of neuronal signaling programs^42^ and also that the invasive cells, to a large extent, depend on transient AMPAR signaling.^29^

Knowing that rBOs contain AMPA receptors, we therefore investigated how AMPA receptor inhibition affected the invasive process. We used the highly invasive BG5 model, which has previously been shown to form synapses with neurons in vivo.^33^ Interestingly, perampanel inhibited single-cell invasion but had a limited effect on cellular competition. This suggests that glioma-neuronal synapses are critical for single-cell invasion into the brain parenchyma, while tissue replacement by tumor cells may be driven by other mechanisms.

In conclusion, our 3D invasion model effectively captures the structural and functional complexity of the differentiated brain tissue, providing a powerful and accessible platform for uncovering GB invasion mechanisms and for identifying new therapeutic vulnerabilities.

## Supplementary Material

noaf271_Supplementary_Data

## Data Availability

The data that support the findings of this study are available from the corresponding author, upon reasonable request. Additional experimental details are provided in the Supplementary material.

## References

[1] Kelley KW , Pașca SP. Human brain organogenesis: toward a cellular understanding of development and disease. Cell. 2022;185:42–61. 34774127 10.1016/j.cell.2021.10.003 PMC13523617

[2] Bjerkvig R. Reaggregation of fetal rat brain cells in a stationary culture system. II: ultrastructural characterization. In Vitro Cell Dev Biol. 1986;22:193–200. 3700322 10.1007/BF02623303

[3] Lancaster MA , Knoblich JA. Generation of cerebral organoids from human pluripotent stem cells. Nat Protoc. 2014;9:2329–2340. 25188634 10.1038/nprot.2014.158 PMC4160653

[4] Farin A , Suzuki SO, Weiker M, Goldman JE, Bruce JN, Canoll P. Transplanted glioma cells migrate and proliferate on host brain vasculature: a dynamic analysis. Glia. 2006;53:799–808. 16541395 10.1002/glia.20334

[5] Madan E , Pelham CJ, Nagane M, et al. Flower isoforms promote competitive growth in cancer. Nature. 2019;572:260–264. 31341286 10.1038/s41586-019-1429-3

[6] de Gooijer MC , Guillén Navarro M, Bernards R, Wurdinger T, van Tellingen O. An experimenter’s guide to glioblastoma invasion pathways. Trends Mol Med. 2018;24:763–780. 30072121 10.1016/j.molmed.2018.07.003

[7] Linkous A , Balamatsias D, Snuderl M, et al. Modeling patient-derived glioblastoma with cerebral organoids. Cell Rep. 2019;26:3203–3211.e3205. 30893594 10.1016/j.celrep.2019.02.063 PMC6625753

[8] Krieger TG , Tirier SM, Park J, et al. Modeling glioblastoma invasion using human brain organoids and single-cell transcriptomics. Neuro Oncol. 2020;22:1138–1149. 32297954 10.1093/neuonc/noaa091 PMC7594554

[9] da Silva B , Mathew RK, Polson ES, Williams J, Wurdak H. Spontaneous glioblastoma spheroid infiltration of early-stage cerebral organoids models brain tumor invasion. SLAS Discov. 2018;23:862–868. 29543559 10.1177/2472555218764623

[10] Goranci-Buzhala G , Mariappan A, Gabriel E, et al. Rapid and efficient invasion assay of glioblastoma in human brain organoids. Cell Rep. 2020;31:107738. 32521263 10.1016/j.celrep.2020.107738

[11] Sarnow K , Majercak E, Qurbonov Q, et al. Neuroimmune-competent human brain organoid model of diffuse midline glioma. Neuro Oncol. 2025;27:369–382. 39561098 10.1093/neuonc/noae245 PMC11812031

[12] Stanchi F , Matsumoto K, Gerhardt H. Imaging glioma progression by intravital microscopy. Methods Mol Biol. 2019;1862:227–243. 30315471 10.1007/978-1-4939-8769-6_16

[13] Bjerkvig R , Tønnesen A, Laerum OD, Backlund EO. Multicellular tumor spheroids from human gliomas maintained in organ culture. J Neurosurg. 1990;72:463–475. 2406382 10.3171/jns.1990.72.3.0463

[14] Sundstrøm T , Daphu I, Wendelbo I, et al. Automated tracking of nanoparticle-labeled melanoma cells improves the predictive power of a brain metastasis model. Cancer Res. 2013;73:2445–2456. 23423977 10.1158/0008-5472.CAN-12-3514

[15] Ivascu A , Kubbies M. Rapid generation of single-tumor spheroids for high-throughput cell function and toxicity analysis. J Biomol Screen. 2006;11:922–932. 16973921 10.1177/1087057106292763

[16] Cox J , Hein MY, Luber CA, Paron I, Nagaraj N, Mann M. Accurate proteome-wide label-free quantification by delayed normalization and maximal peptide ratio extraction, termed MaxLFQ. Mol Cell Proteomics. 2014;13:2513–2526. 24942700 10.1074/mcp.M113.031591 PMC4159666

[17] Jäger C , Hiller K, Buttini M. Metabolic profiling and quantification of neurotransmitters in mouse brain by gas chromatography-mass spectrometry. Curr Protoc Mouse Biol. 2016;6:333–342. 27584556 10.1002/cpmo.15

[18] Golebiewska A , Hau AC, Oudin A, et al. Patient-derived organoids and orthotopic xenografts of primary and recurrent gliomas represent relevant patient avatars for precision oncology. Acta Neuropathol. 2020;140:919–949. 33009951 10.1007/s00401-020-02226-7 PMC7666297

[19] Keunen O , Johansson M, Oudin A, et al. Anti-VEGF treatment reduces blood supply and increases tumor cell invasion in glioblastoma. Proc Natl Acad Sci U S A. 2011;108:3749–3754. 21321221 10.1073/pnas.1014480108 PMC3048093

[20] Zhang Z , Jiao YY, Sun QQ. Developmental maturation of excitation and inhibition balance in principal neurons across four layers of somatosensory cortex. Neuroscience. 2011;174:10–25. 21115101 10.1016/j.neuroscience.2010.11.045 PMC3020261

[21] Maugard M , Vigneron PA, Bolaños JP, Bonvento G. l-Serine links metabolism with neurotransmission. Prog Neurobiol. 2021;197:101896. 32798642 10.1016/j.pneurobio.2020.101896

[22] Yudkoff M , Daikhin Y, Nissim I, et al. Brain amino acid requirements and toxicity: the example of leucine. J Nutr. 2005;135:1531s–1538s. 15930465 10.1093/jn/135.6.1531S

[23] Shi Y , Kirwan P, Livesey FJ. Directed differentiation of human pluripotent stem cells to cerebral cortex neurons and neural networks. Nat Protoc. 2012;7:1836–1846. 22976355 10.1038/nprot.2012.116

[24] Baumert BG , Rutten I, Dehing-Oberije C, et al. A pathology-based substrate for target definition in radiosurgery of brain metastases. Int J Radiat Oncol Biol Phys. 2006;66:187–194. 16814946 10.1016/j.ijrobp.2006.03.050

[25] Semple BD , Blomgren K, Gimlin K, Ferriero DM, Noble-Haeusslein LJ. Brain development in rodents and humans: identifying benchmarks of maturation and vulnerability to injury across species. Prog Neurobiol. 2013;106-107:1–16. 23583307 10.1016/j.pneurobio.2013.04.001 PMC3737272

[26] Giese A , Loo MA, Tran N, Haskett D, Coons SW, Berens ME. Dichotomy of astrocytoma migration and proliferation. Int J Cancer. 1996;67:275–282. 8760599 10.1002/(SICI)1097-0215(19960717)67:2<275::AID-IJC20>3.0.CO;2-9

[27] Ratliff M , Karimian-Jazi K, Hoffmann DC, et al. Individual glioblastoma cells harbor both proliferative and invasive capabilities during tumor progression. Neuro Oncol. 2023;25:2150–2162. 37335907 10.1093/neuonc/noad109 PMC10708941

[28] Osswald M , Jung E, Sahm F, et al. Brain tumour cells interconnect to a functional and resistant network. Nature. 2015;528:93–98. 26536111 10.1038/nature16071

[29] Venkataramani V , Yang Y, Schubert MC, et al. Glioblastoma hijacks neuronal mechanisms for brain invasion. Cell. 2022;185:2899–2917.e31. e2831. 35914528 10.1016/j.cell.2022.06.054

[30] Joseph JV , Magaut CR, Storevik S, et al. TGF-β promotes microtube formation in glioblastoma through thrombospondin 1. *Neuro Oncol*. 2022;24:541–553. 34543427 10.1093/neuonc/noab212 PMC8972291

[31] Seystahl K , Papachristodoulou A, Burghardt I, et al. Biological role and therapeutic targeting of TGF-β(3) in glioblastoma. Mol Cancer Ther. 2017;16:1177–1186. 28377490 10.1158/1535-7163.MCT-16-0465

[32] Taylor KR , Barron T, Hui A, et al. Glioma synapses recruit mechanisms of adaptive plasticity. Nature. 2023;623:366–374. 37914930 10.1038/s41586-023-06678-1 PMC10632140

[33] Venkataramani V , Tanev DI, Strahle C, et al. Glutamatergic synaptic input to glioma cells drives brain tumour progression. Nature. 2019;573:532–538. 31534219 10.1038/s41586-019-1564-x

[34] Moscona A , Moscona H. The dissociation and aggregation of cells from organ rudiments of the early chick embryo. J Anat. 1952;86:287–301. 12980879 PMC1273752

[35] Qian X , Song H, Ming GL. Brain organoids: advances, applications and challenges. Development. 2019;146:dev166074. 10.1242/dev.166074 PMC6503989 30992274

[36] Madhavan M , Nevin ZS, Shick HE, et al. Induction of myelinating oligodendrocytes in human cortical spheroids. Nat Methods. 2018;15:700–706. 30046099 10.1038/s41592-018-0081-4 PMC6508550

[37] Linkous A , Fine HA. Generating patient-derived gliomas within cerebral organoids. STAR Protoc. 2020;1:100008. 33103125 10.1016/j.xpro.2019.100008 PMC7580125

[38] Zeiss CJ. Comparative milestones in rodent and human postnatal central nervous system development. Toxicol Pathol. 2021;49:1368–1373. 34569375 10.1177/01926233211046933

[39] Mansour AA , Gonçalves JT, Bloyd CW, et al. An in vivo model of functional and vascularized human brain organoids. Nat Biotechnol. 2018;36:432–441. 29658944 10.1038/nbt.4127 PMC6331203

[40] Giese A , Bjerkvig R, Berens ME, Westphal M. Cost of migration: invasion of malignant gliomas and implications for treatment. J Clin Oncol. 2003;21:1624–1636. 12697889 10.1200/JCO.2003.05.063

[41] Monteiro AR , Hill R, Pilkington GJ, Madureira PA. The role of hypoxia in glioblastoma invasion. Cells. 2017;6:45. 29165393 10.3390/cells6040045 PMC5755503

[42] Varn FS , Johnson KC, Martinek J, GLASS Consortium, et al. Glioma progression is shaped by genetic evolution and microenvironment interactions. Cell. 2022;185:2184–2199.e16. 35649412 10.1016/j.cell.2022.04.038 PMC9189056

[43] Eskilsson E , Rosland GV, Talasila KM, et al. EGFRvIII mutations can emerge as late and heterogenous events in glioblastoma development and promote angiogenesis through SRC activation. Neuro Oncol. 2016;18:1644–1655. 27286795 10.1093/neuonc/now113 PMC5791772

