# Supplementary material for: Development of a highly differentiated rat brain organoid model for exploring glioblastoma invasion dynamics and therapy
Source: Neuro Oncol. 2025 Nov 24;28(3):626–42. doi: 10.1093/neuonc/noaf271 (PMC13070497; doi:10.1093/neuonc/noaf271)
Supplement: noaf271_Supplementary_Data [file noaf271_supplementary_data.zip › Supplementary Table 1_Reagents_Resources_rev.docx]

**Supplementary Table 1. Reagents and Resource**

| **REAGENT or RESOURCE** | **SOURCE** | **IDENTIFIER** |
| --- | --- | --- |
| **Antibodies** | | |
| Anti-alpha Tubulin rabbit pAb | Cell Signaling Technology | 2144 |
| Anti-CD11b/ITGAM (D6X1N) Rabbit mAb | Cell Signaling Technology | 49420 |
| Anti-CD68, clone ED1, Mouse mAb | BIO-RAD | MCA3418 |
| Anti-GFAP (D1F4Q) XP® Rabbit mAb | Cell Signaling Technology | 12389 |
| Anti-GFAP (GA5) Mouse mAb | Cell Signaling Technology | 3670 |
| Anti-Iba1 antibody [1022-5] Mouse mAb | Abcam | ab15690 |
| Anti-Ki67 Antigen MIB-5 [Rat] Mouse mAb | Dako | M7248 |
| Anti-MBP Rabbit Antibody (C-16), pAb | Santa Cruz | sc-13914 |
| Anti-Nestin Antibody [human], clone 10C2 Mouse mAb | Merck Millipore | MAB5326 |
| Anti-Nestin antibody [Rat-401] Mouse mAb | Abcam | ab6142 |
| Anti-NeuN Mouse mAb, clone A60 | Merck Millipore | MAB377 |
| Anti-Olig2 Rabbit mAb [EPR2673] | Abcam | ab109186 |
| Anti-pSmad2/3 (Ser423/425) | Santa Cruz | sc-11769 |
| Anti-Smad2/3 antibody | R&D Systems | AF3797 |
| Anti-Sox2 (D1C7J) XP® Rabbit mAb | Cell Signaling Technology | 14962 |
| Anti-TGFbeta | Abcam | ab1279 |
| Anti-Tubulin β 3 (TUBB3) Antibody | Covance | 801201 (Previously MMS-435P) |
| Goat anti-Mouse IgG (H+L) Cross-Adsorbed Secondary Antibody, Alexa Fluor 647 | Invitrogen | A21235 |
| Goat anti-Mouse IgG (H+L) Secondary Antibody, HRP, pAb | Invitrogen | 31430 |
| Goat anti-Mouse IgG2a, Human ads-FITC | SouthernBiotech | 1080-02 |
| Goat anti-Rabbit IgG (H+L) Cross-Adsorbed Secondary Antibody, Alexa Fluor 647 | Invitrogen | A21244 |
| Goat anti-rabbit IgG (H+L) Cross-Adsorbed Secondary Antibody, pAb | Invitrogen | 31462 |
| Goat anti-Rabbit IgG Antibody (H+L), Biotinylated | Vector Laboratoires | BA-1000 |
| Horse anti-Mouse IgG Antibody, rat adsorbed (H+L), Biotinylated | Vector Laboratoires | BA-2001 |
| Anti-Carbonic Anhydrase IX/CA9 | Proteintech | 11071-1-AP |
| Anti-BNIP3 | Proteintech | 68091-1-Ig |
| Anti-ß-Actin | Proteintech | 81115-1-RR |
| **Chemicals and reagents** | | |
| 1,4-Dithiothreitol (DTT) | Sigma-Aldrich | D9779 |
| 3,3'-diaminobenzidine (DAB) chromogen | Dako | K4065 |
| 4 % paraformaldehyde (PFA) | Sigma-Aldrich | P6148 |
| 4′,6-diamidino-2-phenylindole (DAPI) | Invitrogen | D1306 |
| Accutase | Stemcell Technologies | 07920 |
| Acetonitrile | Sigma-Aldrich | 900667 |
| B-27™ Supplement (50X), serum free | Gibco | 17504-044 |
| Bovine serum albumin (BSA) | Sigma-Aldrich | 810531 |
| Chloroform | Sigma-Alrich | 650498 |
| cOmplete™ Protease Inhibitor Cocktail | Roche | 11697498001 |
| Difco Agar Noble | BD Biosciences | 214230 |
| Dulbecco′s Modified Eagle′s Medium - high glucose | Sigma-Aldrich | D5648 |
| EGF | R&D Systems | 236-EG |
| Entellan | Sigma-Aldrich | 1.07960 |
| Essential 8 Media | Gibco | A1517001 |
| Fetal Bovine Serum, heat-inactivated | Gibco | A5256801 |
| FGF | Peprotech | 100-18B |
| Formalin, 10 % | CellStor | BAF-0010-01Y |
| Formic acid | Sigma-Aldrich | 5.33002 |
| Gadodiamide (Omniscan) | GE Healthcare, Stockholm, Sweden | 00407069010 |
| Geltrex | Gibco | A1413301 |
| Glutaraldehyde | Sigma-Aldrich | 3802 |
| Halt™ Protease Inhibitor Cocktail, EDTA-Free (100X) | ThermoFisher Scientific | 87785 |
| Harris-Hematoxylin | CellPath | RBA-4213-00A |
| Heparin 5000 IU/ml | Leo Pharma | 464327 |
| HRP ABC reagent | Vector Laboratories | PK-4000 |
| Hydrochloric acid solution (incl. dopamine-D4) | C/D/N Isotopes Inc. |  |
| Hydrogen peroxide solution (H_2_O_2_), 30 % | Sigma-Aldrich | H1009 |
| Iodoacetamide | Sigma-Aldrich | I6125 |
| Isoflurane | Sigma-Aldrich | 792632 |
| L-Glutamine (200 mM) | Lonza | BW17-605E |
| Lentiviral particles from pCDH-CMV-MCS-EF1α-copGFP cDNA Dual Promoter Cloning and Expression Lentivector | System Biosciences | CD511B-1 |
| Lentiviral particles from rLV.EF1. Zs Green 1-9, rLV.EF1. AMCyan 1-9; rLV.EF1. td Tomato 1-9 | Vectalys TM | 0038VCT, 0039VCT, 0036VCT |
| Lys-C | Wako | 121-05063 |
| Matrigel | Corning | 356234 |
| MEM Eagle Nonessential Amino Acid Solution (100X) | Lonza | 13114E |
| Methoxyamine hydrochloride | Sigma-Aldrich | 89803 |
| N-methyl-N-trimethylsilyl-trifluoroacetamide (MSTFA) | Macherey-Nagel | 701270.110 |
| Neurobasal Medium | Gibco | 21103049 |
| NuPAGE™ 10%, Bis-Tris, 1.0–1.5 mm, Mini Protein Gels | Invitrogen | NP0301BOX |
| Osmium tetroxide (OsO_4_) | Sigma-Aldrich | 419494 |
| Penicillin/Streptomycin | Gibco | 15140122 |
| Pentanedioic-d6 acid | C/D/N Isotopes Inc. | D-5227 |
| Perampanel | Ad00Q | A12498 |
| Phosphate Buffered Saline (10X) (PBS) | Lonza | BW17-517Q |
| Plasmocin | InvivoGen | ant-mpp |
| poly(lactide-co-glycolide) braided fibers, size 5-0 | Ethicon | J503G |
| PVDF membrane | BioRad | 1620177 |
| Pyridine | Sigma-Aldrich | 270407 |
| RIPA Lysis and Extraction Buffer | ThermoFisher Scientific | 89901 |
| Sodium chloride (NaCl) | Sigma-Aldrich | S5886 |
| Sodium deoxycholate (SDC) | Sigma-Aldrich | 264103 |
| StemPro Accutase | Gibco | A1110501 |
| SuperSignal West Pico PLUS Chemiluminescent Substrate | ThermoFisher Scientific | 34580 |
| TGF-β | PeproTech | 100-21 |
| TGF-β inhibitor LY2157299 | Peprotech | 7007224 |
| Tridecanoic-d25 acid | C/D/N Isotopes Inc. | D-4002 |
| Trifluoroacetic acid (TFA) | Sigma-Aldrich | 80457 |
| TrisHCl | Sigma-Aldrich | T3253 |
| Trypsin | Promega | V5280 |
| Trypsin/EDTA | Lonza | CC-5012 |
| U-^13^C_5_ ribitol | Omicron Biochemicals | ALD-062 |
| **Critical commercial assays** | | |
| EZQ Protein Quantitation Kit | Invitrogen | R33200 |
| hPSC Genetic Analysis Kit | Stemcell Technologies | 07550 |
| miRNeasy Mini Kit | Qiagen | 217004 |
| Pierce BCA Protein Assay Kit | ThermoFisher Scientific | 23225 |
| TruSeq Stranded Total RNA Library Prep Kit | Illumina | 20020597 |
| ACS Neural Tissue Dissociation Kit (P) | Miltenyi | 130-092-628 |
| The SMART-Seq v4 Ultra Low Input RNA Kit | TaKaRa | 634894 |
