# Supplementary material for: Development of a highly differentiated rat brain organoid model for exploring glioblastoma invasion dynamics and therapy
Source: Neuro Oncol. 2025 Nov 24;28(3):626–42. doi: 10.1093/neuonc/noaf271 (PMC13070497; doi:10.1093/neuonc/noaf271)
Supplement: noaf271_Supplementary_Data [file noaf271_supplementary_data.zip › Supplementary Figures_rev.pdf]

Supplementary Figures

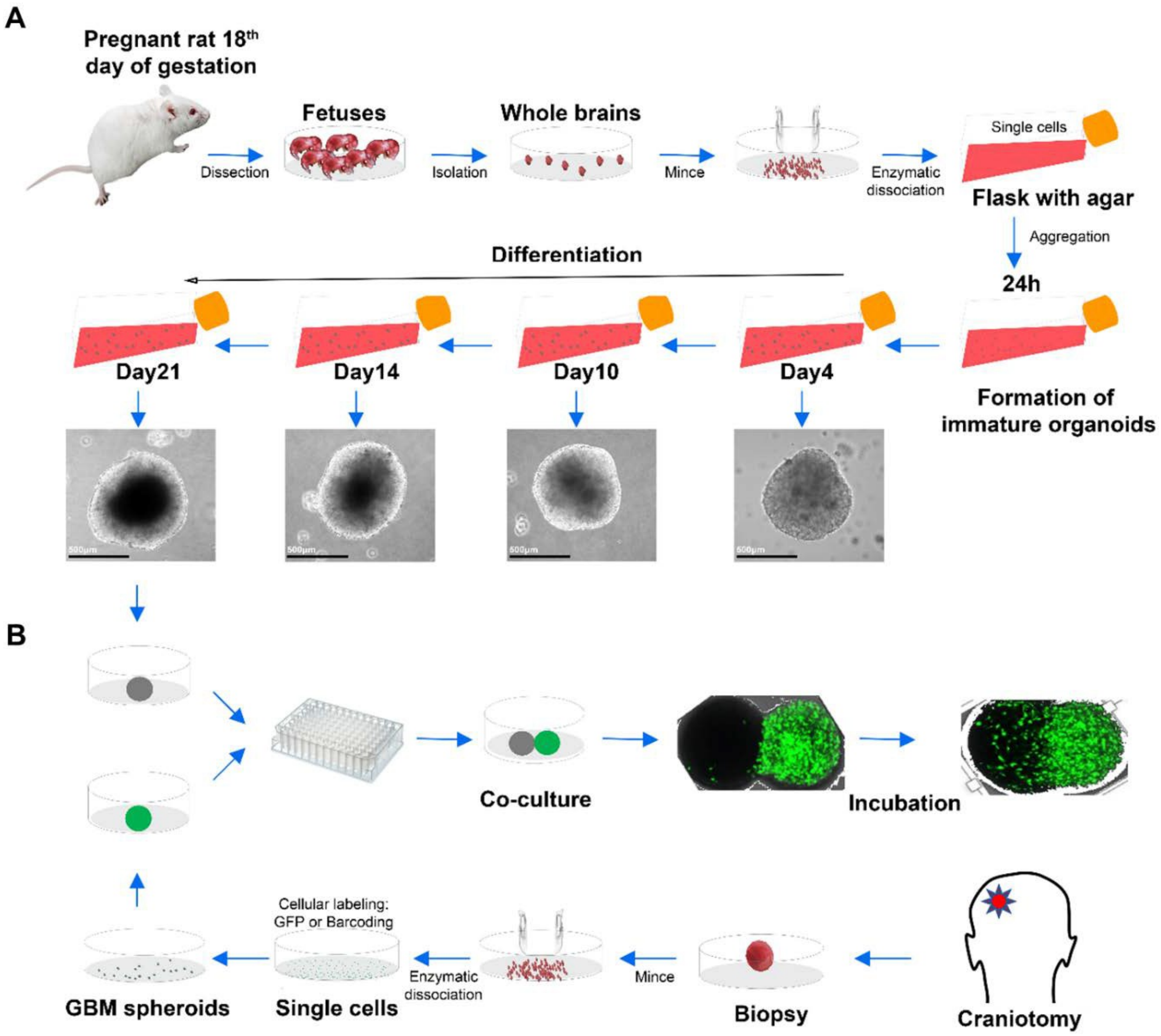

Supplementary Fig. 1. Schematic presentation of the *ex-vivo* rBO-human GBM organoid confrontation system.

A) Fetal rat brains are collected at the 18th day of gestation whereupon they are dissociated into a single cell suspension. The cells are then reaggregated, in a medium agar overlay culture, into immature organoids. Following a 21-day culture period, the immature reaggregated neural cells (Day 4) undergo a reorganization and an extensive cellular differentiation into mature, highly organized rat brain organoids (rBOs).

B) The establishment of human GBM organoids: Human GBM biopsies are minced and dissociated into a single cell suspension, whereupon the dissociated cells are labeled by lentiviral transfection, with GFP, or with RFP, GFP and CYAN (see Supplementary Fig. 12). Following cell culture, tumor organoids are formed. These are confronted with the rBOs. Tumor cell invasion into the rBOs is then monitored in real time.

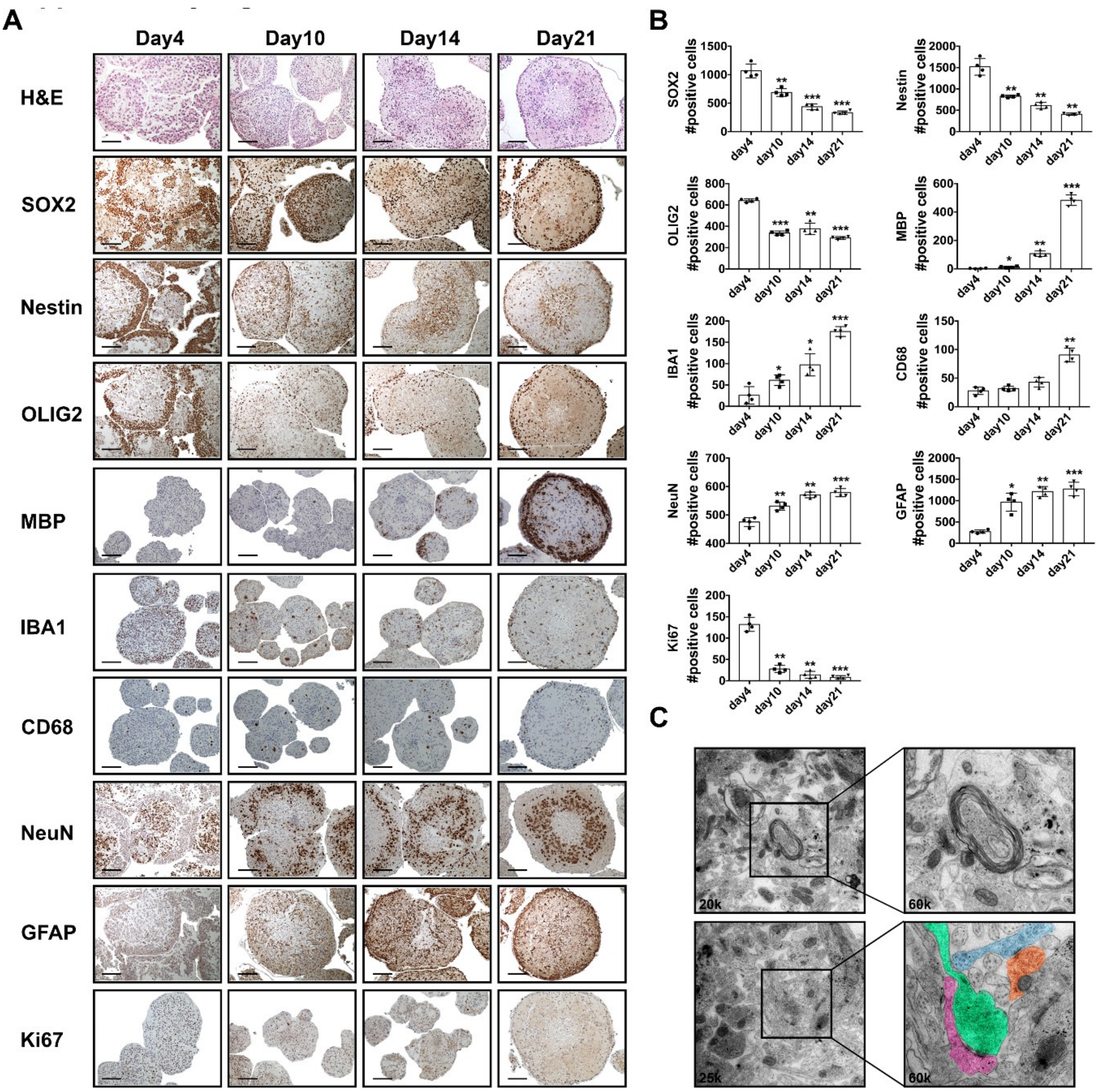

Supplementary Fig. 2. Immunostaining of neural and brain differentiation markers during rBO differentiation.

A) Immunostaining of neural cells and differentiation markers during rBO development (day 4, 10, 14 and 21) showing an extensive cellular reorganization during organoid differentiation (Scale bar = 100 µm).

B) Immunohistochemical quantification showing a significant increase in differentiation markers (MPB, IBA1, CD68, NeuN, GFAP) and a reduction in markers associated with early brain development (SOX2, Nestin, OLIG2), as well as the Ki67 cell proliferation marker (One-way ANOVA and Bonferroni post hoc were used to perform comparative statistical analysis between the two groups). Each dot represents independent experiments of individual rBOs. * p<0.05, ** p<0.01, *** p<0.001).

C) Upper panels: Transmission electron micrographs from a 21-day-old rBO showing a myelinated axon within the neuropil as well as the presence of multiple synapses (lower panel, outlined in color).

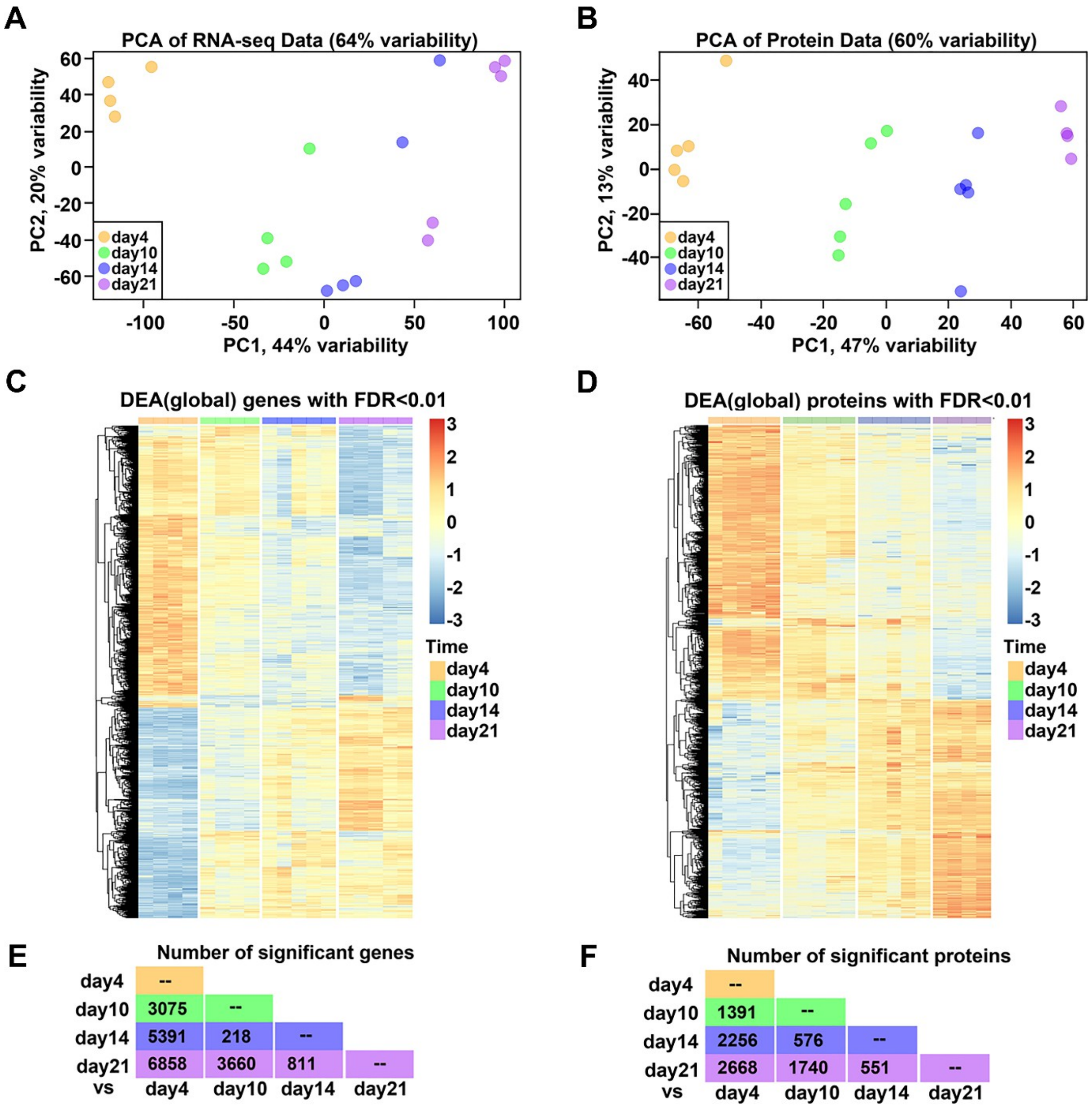

Supplementary Fig. 3. Analyses of global gene and protein expression data during rBO differentiation.

A) PCA analysis of global gene expression data obtained at day 4, 10, 14, and 21 of rBO differentiation, showing a clear separation between the different time points. Each dot represents independent experiments (n=4, except for day 21, where n=4).

B) PCA analysis of global protein expression data at day 4, 10, 14 and 21 of rBO differentiation, showing a clear separation between time points.

C) Heatmap of global gene expression data at day 4, 10, 14, and 21 of rBO differentiation.

D) Heatmap of global protein expression data at day 4, 10, 14, and 21 of rBO differentiation.

E) Number of significant genes differentially expressed between the different time points

F) Number of significant proteins differentially expressed between the different time points.

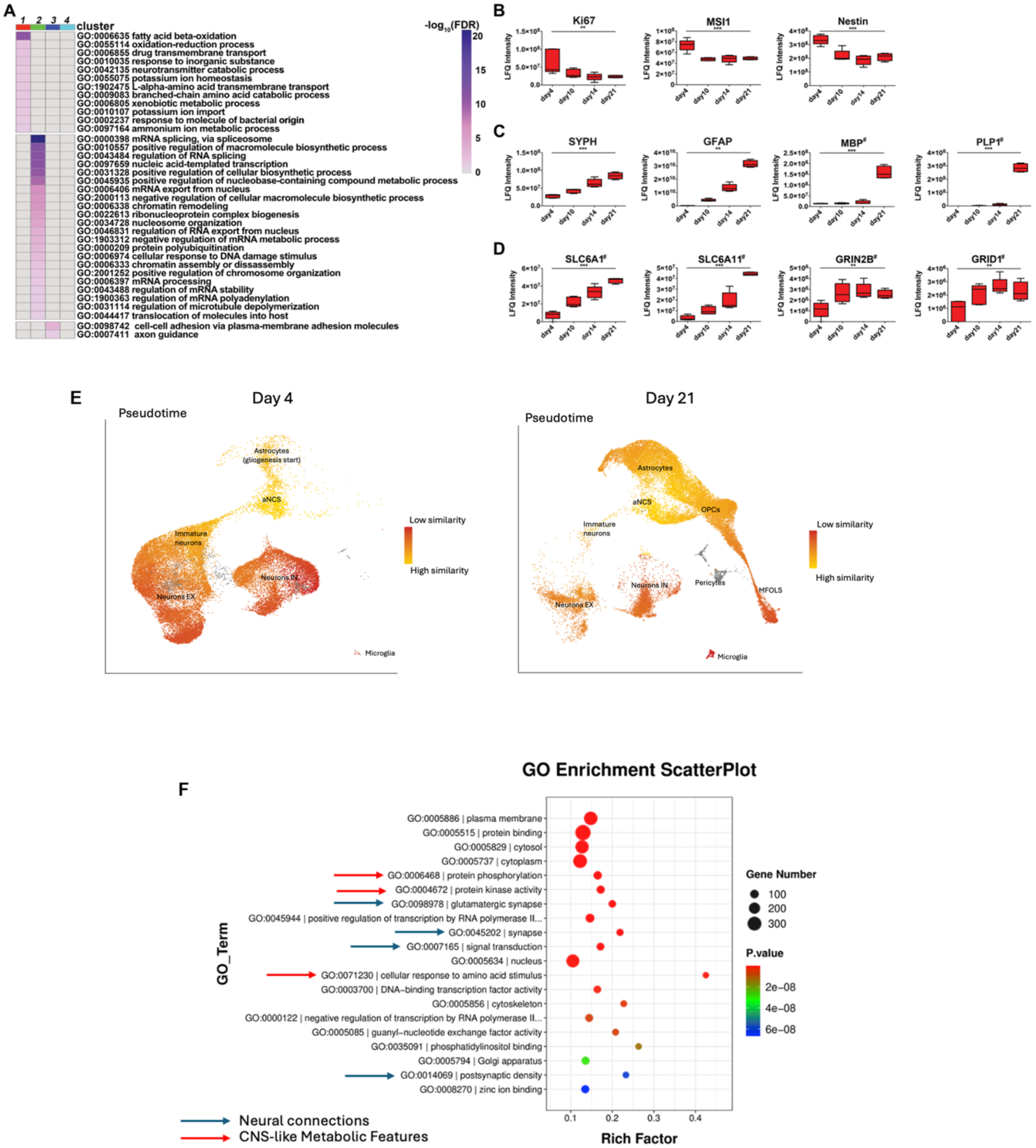

Supplementary Fig. 4. Gene ontology and proteomic expression during rBO differentiation.

A) The Gene Ontology for cluster 1 reveals a significant upregulation of biological processes related to neurotransmitter uptake and catabolic processes, as well as pathways crucial for glial ion and neural network homeostasis. Cluster 2 shows a downregulation of numerous biological processes associated with early organ development between day 4 and 21.

B) The proteomics analysis reveals that cell proliferation (as indicated by Ki67), stem cell markers (Musashi-1; MSI1), and Nestin are significantly reduced from day 4 to day 21.

C) In contrast, the mature neuronal marker SYPH and the astrocyte marker GFAP, as well as the oligodendrocyte differentiation markers MBP and PLP1, are increased.

D) The GABA transporters SLC6A1 and SLC6A11, known to be involved in the rapid removal of GABA, were increased between 4 and 21 days. Additionally, the glutamate receptor proteins

GRIN2B and GRID1 exhibited a significant increase during the same period.

E) Pseudotime trajectory of the single-cell transcriptomes colored by similarity score. Cells are arranged along pseudotime to represent transcriptional progression inferred from sequencing data. Each point corresponds to a single cell, with colors indicating the degree of transcriptional similarity (yellow = high similarity, red = low similarity). The trajectory reveals distinct transcriptional states and transitions between cell populations along the inferred developmental continuum.

F) Gene-Ontology terms derived from the single-cell data pointing at neuronal connections (blue arrows) and CNS-like metabolic features (red arrows).

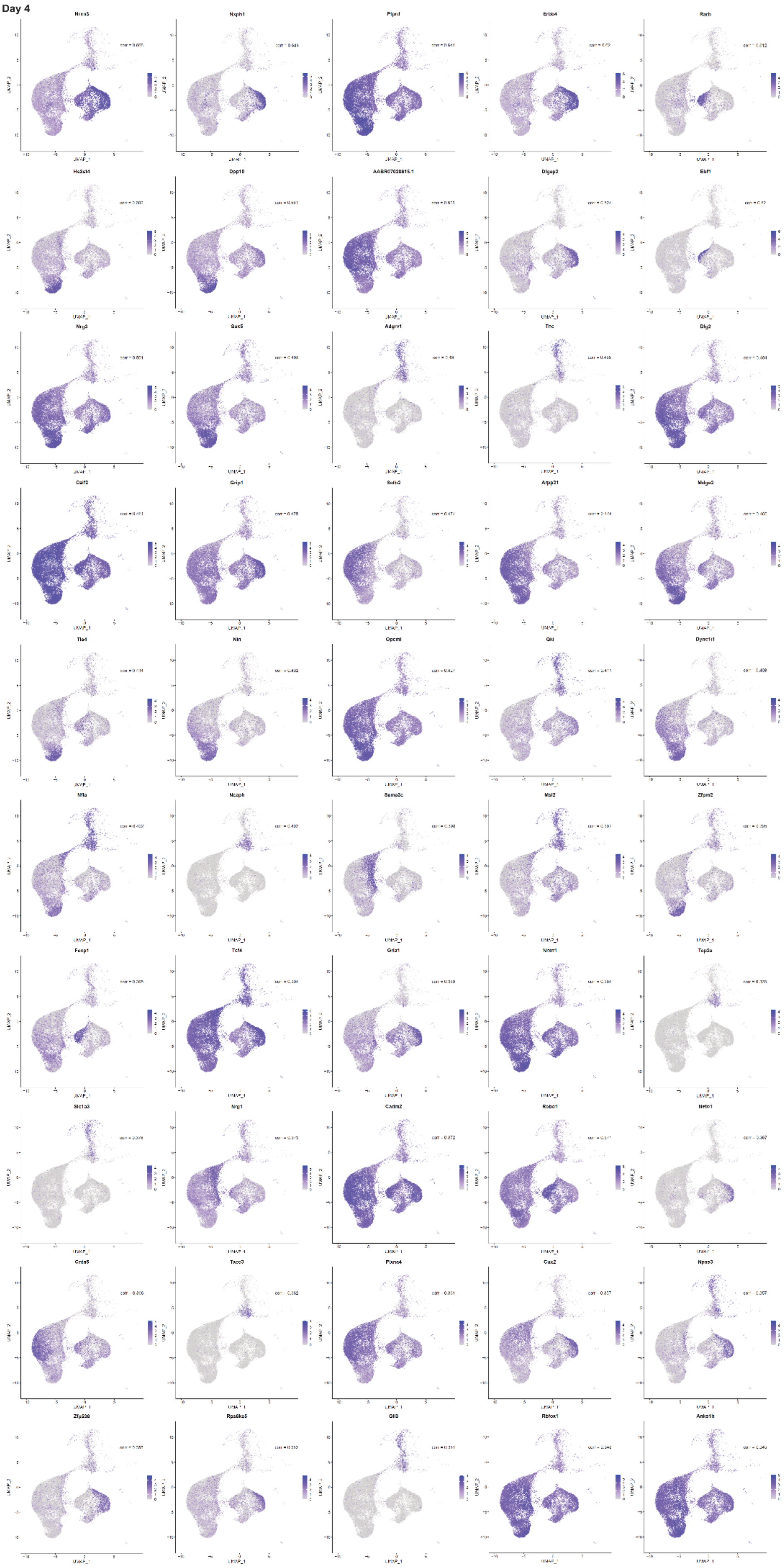

Supplementary Fig. 5. The 50-gene signature used to identify cell lineage allocation in 4-day-old rBOs.

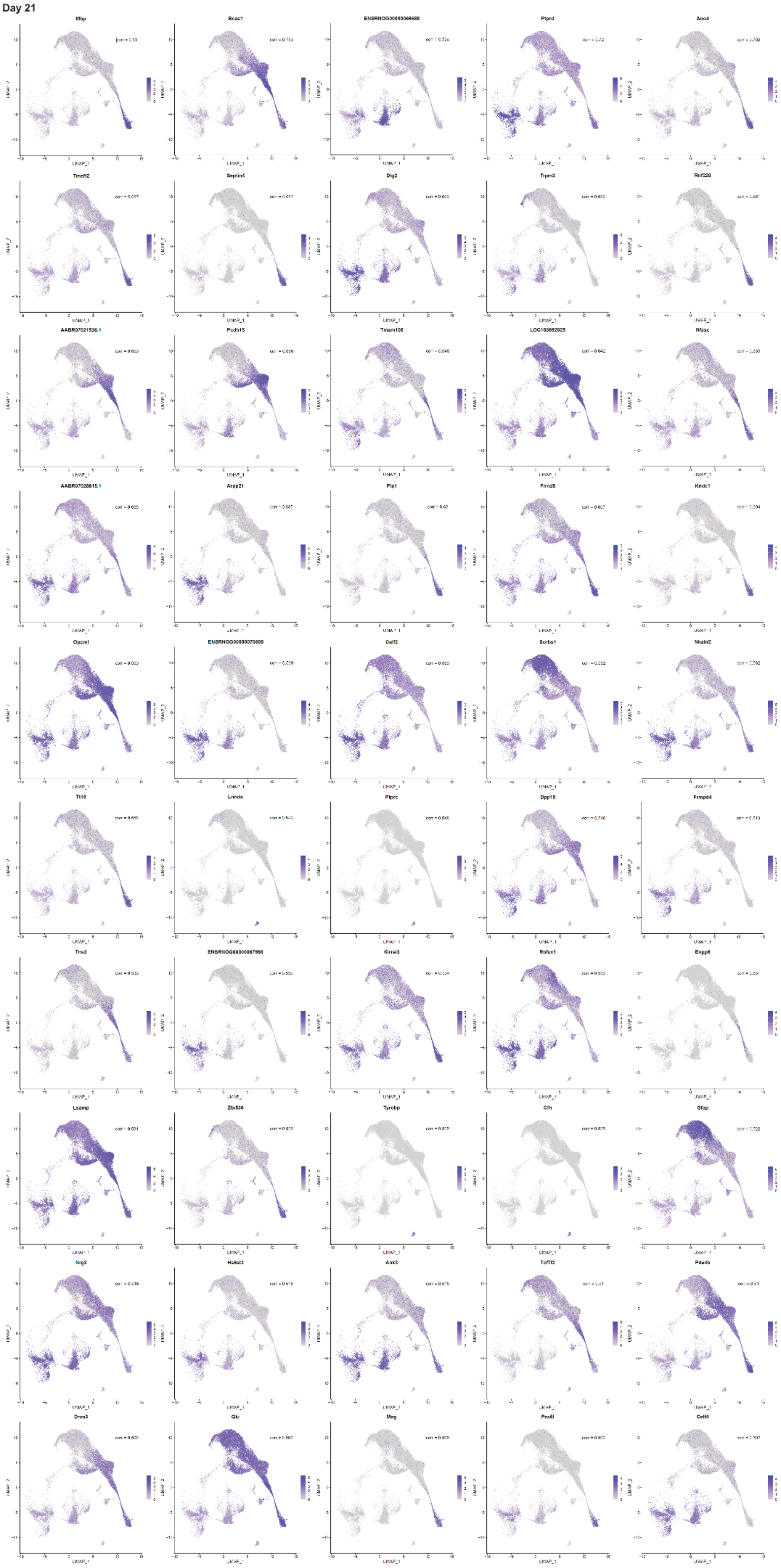

Supplementary Fig. 6. The 50-gene signature used to identify cell lineage allocation in 21-day-old rBOs.

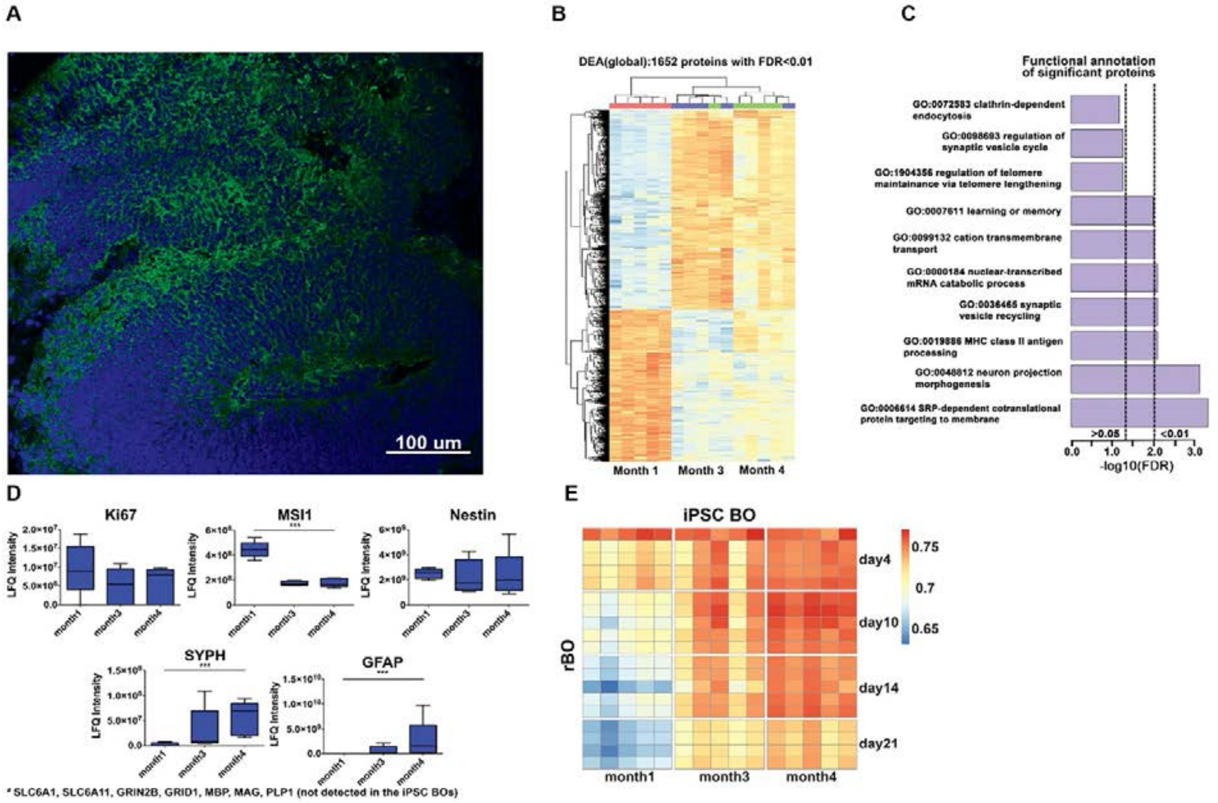

Supplementary Fig. 7. Gene ontology and proteomic expression during human iPSC BO differentiation.

A) Immunofluorescence of TUBB3 expression in a sectioned 3-month-old iPSC organoid. TUBB3 is known to be involved in neurogenesis and axon guidance as well as maintenance.

B) Data envelopment (DEA) hierarchical clustering of proteins identified in the iPSC BOs shows a clear separation between organoids at 1, 3, and 4 months of culture, with the largest separation between months 1 and 3 (n=5).

C) Gene ontology analysis reveals a significant upregulation of biological processes associated with brain development.

D) Proteomics analysis showing the expression of Ki67, MSI1, Nestin, SYPH and GFAP in iPSC organoids at different stages of differentiation. SLC&A1, SLC6A11, GRIN2B, MBP, MAG, and PLP1 were not detected in the iPSCs.

E) Heatmap showing the Pearson correlation coefficient between the protein expression observed in both proteomics studies of rBOs and human iPSC BOs (n=5 for each time point except for rBOs at day 21 where n=4). The protein expression profiles from 4-month-old iPCs BOs corresponded more to rBOs at day 10; whereas the highest correlation of 1 1-month-old iPSC BOs corresponded to 4-day-old rBOs.

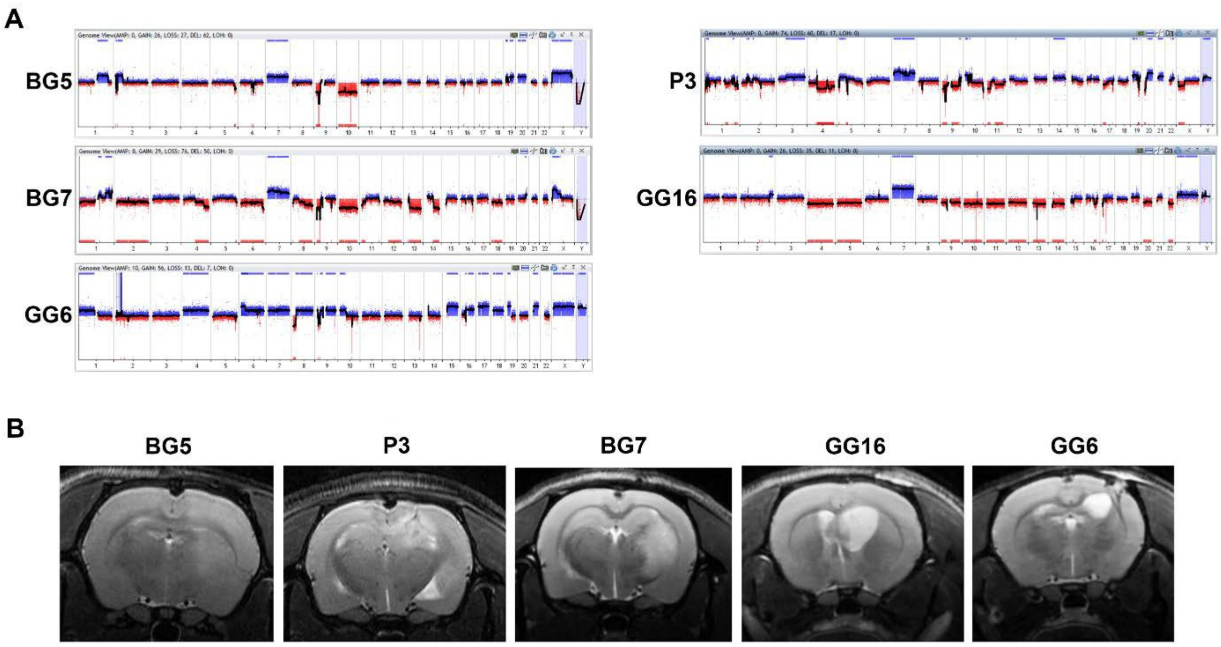

Supplementary Fig. 8. DNA copy number alterations and MRI imaging show typical GBM features of the tumor models used.

A) Similar DNA copy number alterations (CNAs) as seen in human isocitrate dehydrogenase (*IDH*) wild-type GBMs.

B) High-resolution 7T MRI showing different contrast enhancement and invasive growth patterns *in vivo.*

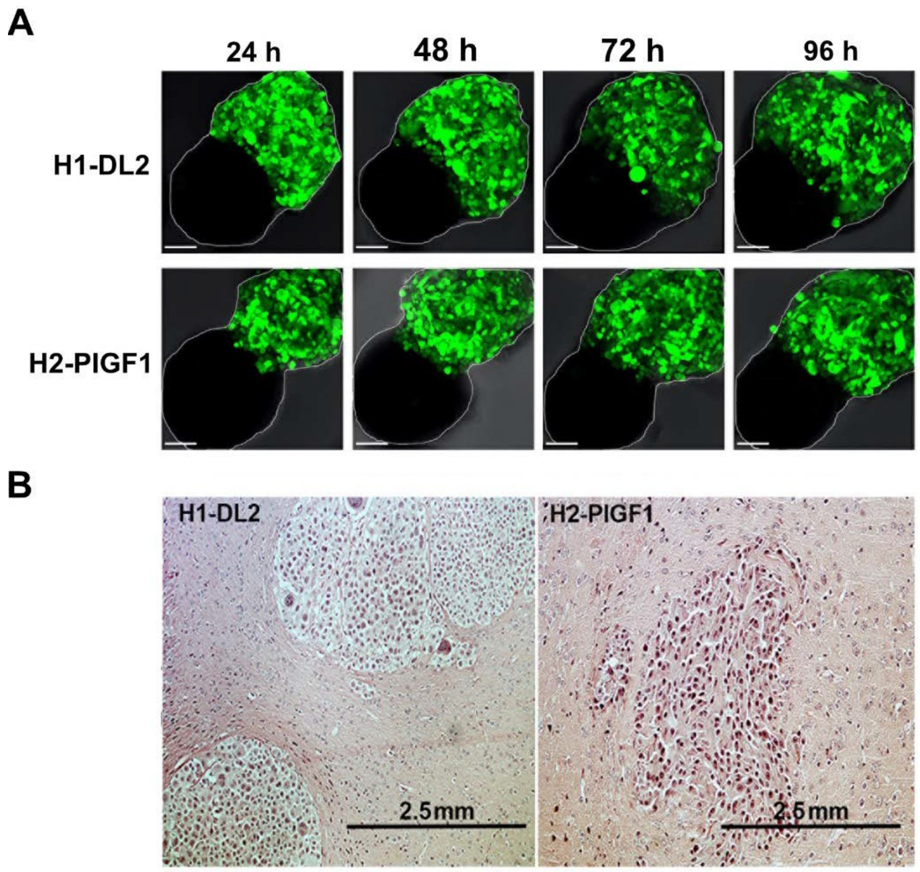

Supplementary Fig. 9. Two human melanoma metastasis models show little to no invasion into the rBOs as well as in vivo.

A) Co-cultures, using two human brain metastasis models do not display any invasion into the rBOs.

B) Histological sections of brain metastases developed after intracardiac delivery in vivo also show little to no invasion within the brain.

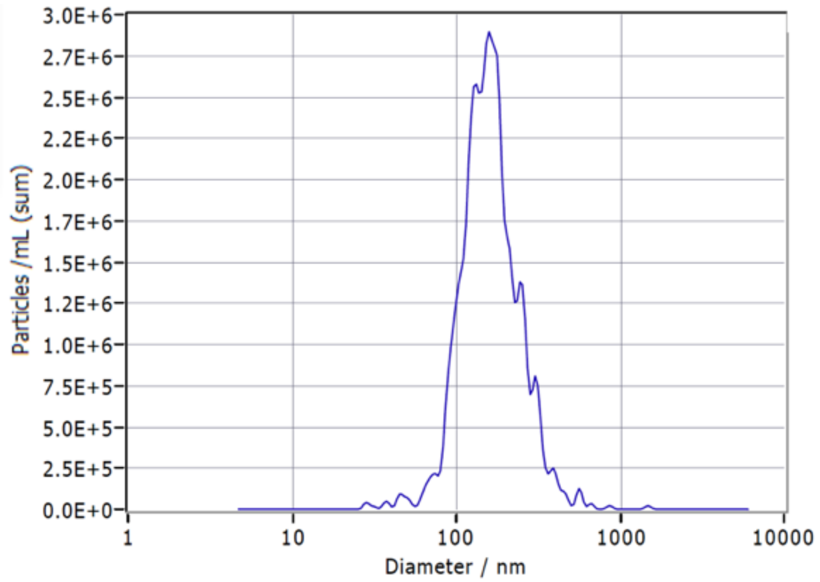

Supplementary Fig. 10. Characterization of EVs secreted by rBOs.
Nanoparticle tracking analysis (NTA) of extracellular vesicles. The graph shows the particle size distribution, expressed as particle concentration (particles/ml) versus diameter (nm, logarithmic scale). A predominant population of vesicles is observed with diameters centered around ~150 nm, consistent with the expected size range for extracellular vesicles. Measurements were performed using a Zeta Potential and Particle Size Analyzer (Model: Zeta PALS, Bruker, Germany).

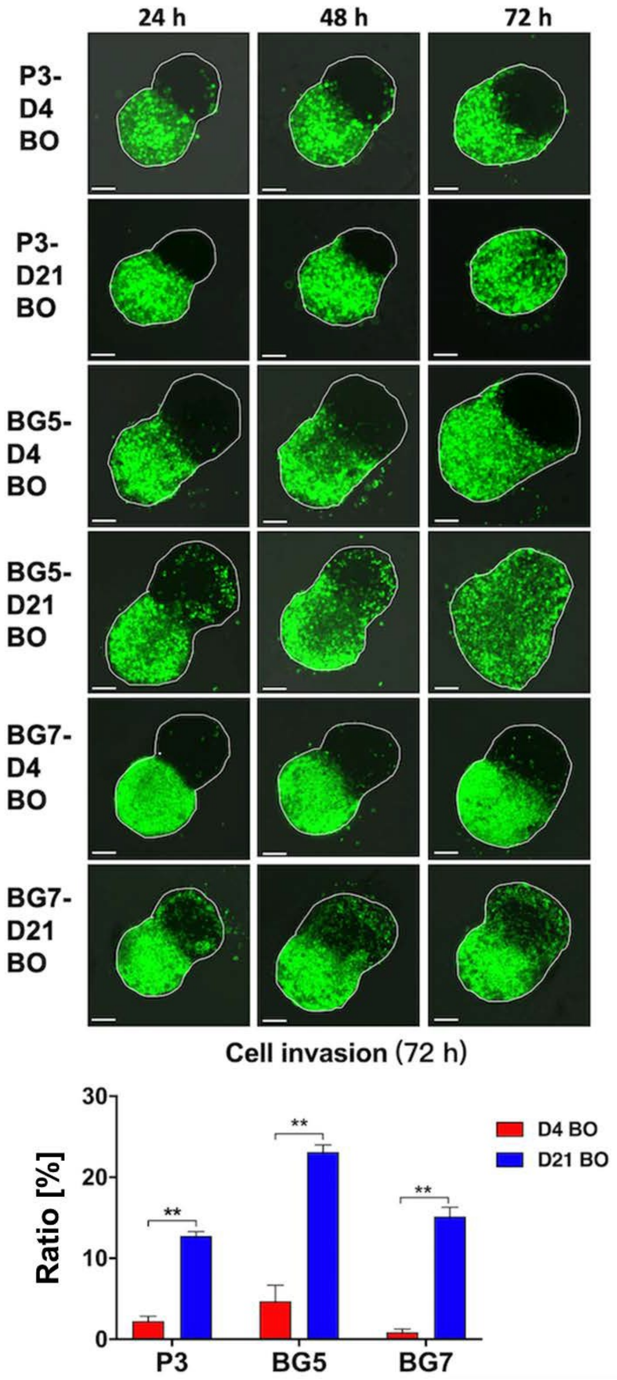

Supplementary Fig. 11. Comparison of invasion between immature 4-day-old rBOs 4 and 21-day-old rBOs.

Upper panel: Comparative experiment showing invasion of P3, BG5 and BG7 into 4-day-old rBOs and into 21-day-old rBOs. Very little invasion is observed in the 4-day-old rBOs, indicating that the differentiation status of the BOs is crucial in assessing invasion parameters.

Lower panel: Quantification of invasion showing a significant reduction of invasion in 4-day-old rBOs compared to differentiated organoids ± SEM (** $p<0.01$, $n=3$, unpaired t-test)

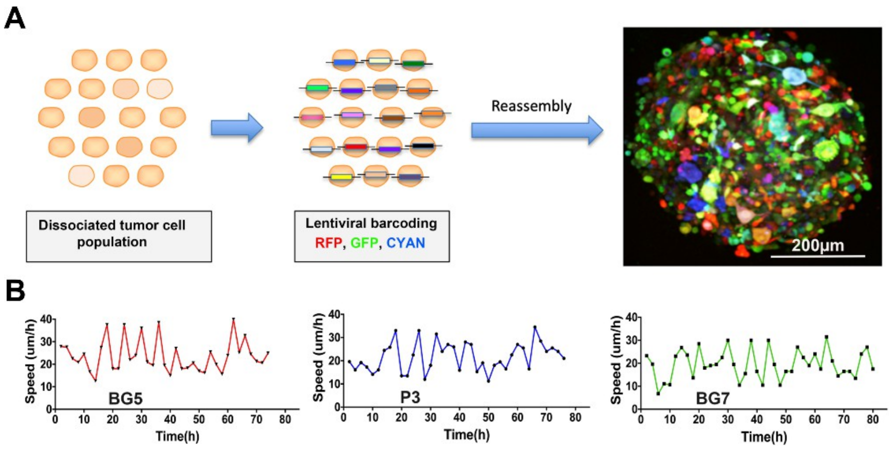

Supplementary Fig. 12. Quantification of invasion and cellular barcoding.

A) For cellular barcoding, GBM organoids are dissociated into a single cell population that is transduced with lentiviral particles expressing fluorescent protein genes (RFP, GFP and CYAN). Following transduction, new organoids are established.

B) Examples of the speed of a single tumor cell (from BG5, P3, and BG7) within the rBOs.
